# Outlier Analysis Defines Zinc Finger Gene Family DNA Methylation in Tumors and Saliva of Head and Neck Cancer Patients

**DOI:** 10.1371/journal.pone.0142148

**Published:** 2015-11-06

**Authors:** Daria A. Gaykalova, Rajita Vatapalli, Yingying Wei, Hua-Ling Tsai, Hao Wang, Chi Zhang, Patrick T. Hennessey, Theresa Guo, Marietta Tan, Ryan Li, Julie Ahn, Zubair Khan, William H. Westra, Justin A. Bishop, David Zaboli, Wayne M. Koch, Tanbir Khan, Michael F. Ochs, Joseph A. Califano

**Affiliations:** 1 Department of Otolaryngology—Head and Neck Surgery, Johns Hopkins Medical Institutions, Baltimore, Maryland, United States of America; 2 Department of Urology, Northwestern University, Chicago, Illinois, United States of America; 3 Division of Oncology Biostatistics, Department of Oncology, Johns Hopkins Medical Institutions, Baltimore, Maryland, United States of America; 4 Department of Statistics, The Chinese University of Hong Kong, Shatin, NT, Hong Kong SAR, China; 5 University of Virginia, Department of Pathology, Charlottesville, Virginia, United States of America; 6 Department of Pathology, Johns Hopkins Medical Institutions, Baltimore, Maryland, United States of America; 7 Department of Mathematics and Statistics, The College of New Jersey, Ewing, New Jersey, United States of America; 8 Milton J. Dance Head and Neck Center, Greater Baltimore Medical Center, Baltimore, Maryland, United States of America; 9 Division of Otolaryngology / Head and Neck Surgery, Department of Surgery, University of California, San Diego, La Jolla, California, United States of America; The Chinese University of Hong Kong, HONG KONG

## Abstract

Head and Neck Squamous Cell Carcinoma (HNSCC) is the fifth most common cancer, annually affecting over half a million people worldwide. Presently, there are no accepted biomarkers for clinical detection and surveillance of HNSCC. In this work, a comprehensive genome-wide analysis of epigenetic alterations in primary HNSCC tumors was employed in conjunction with cancer-specific outlier statistics to define novel biomarker genes which are differentially methylated in HNSCC. The 37 identified biomarker candidates were top-scoring outlier genes with prominent differential methylation in tumors, but with no signal in normal tissues. These putative candidates were validated in independent HNSCC cohorts from our institution and TCGA (The Cancer Genome Atlas). Using the top candidates, *ZNF14*, *ZNF160*, and *ZNF420*, an assay was developed for detection of HNSCC cancer in primary tissue and saliva samples with 100% specificity when compared to normal control samples. Given the high detection specificity, the analysis of ZNF DNA methylation in combination with other DNA methylation biomarkers may be useful in the clinical setting for HNSCC detection and surveillance, particularly in high-risk patients. Several additional candidates identified through this work can be further investigated toward future development of a multi-gene panel of biomarkers for the surveillance and detection of HNSCC.

## Introduction

Head and Neck Squamous Cell Carcinoma (HNSCC) affects an estimated 60,000 individuals in the United States and 600,000 individuals worldwide annually [1, 2]. HNSCC is commonly caused by tobacco and alcohol exposure, as well as by human papillomavirus (HPV) [1]. Despite advances in the understanding of HNSCC biology, approximately half of all patients with HNSCC succumb to the disease within five years of diagnosis [2–4].

It is now widely observed that whole-genome hypomethylation, accompanied by gene-specific promoter hypermethylation, is a general characteristic of solid tumors [5, 6]. Promoter hypermethylation has been described for an extensive number of genes, and commonly results in tumor-suppressor gene transcriptional repression [3]. Given the high rate of DNA methylation abnormalities in cancerous cells as well as DNA methylation signal stability during cell division, the detection of DNA methylation is a valuable tool in the development of biomarkers for cancer detection and prognosis [7–9]. Several whole-genome methylation assays have been performed to define the DNA methylation signature of HNSCC [5, 10–14]. DNA methylation of *DCC*, *EDNRB*, *DAPK*, *CCNA1*, *p16*, *HOXA9* and *KIF1A* genes have been detected in primary tissues and biological fluids, including saliva and plasma [7, 10, 15, 16], derived from HNSCC patients. DNA methylation of several other genes, including *MINT31*, *MGMT*, *NID2*, *ERCC1*, and *TIMP3*, have been proposed as biomarkers of HNSCC that can be detected in a patient’s biofluids [11, 16]. Other genes, like *CYGB*, *RASSF1A*, *SPARC*, *GSTM1*, *cyclinA1*, *MX1*, *WIF1*, *GNG7*, *CYP1A1*, *ZNF132*, *ZNF154*, and *ZNF447* have demonstrated high rates of DNA methylation in primary HNSCC tumors [13, 14, 16–23] and are candidates for further validation of DNA methylation detection in body fluids. Genome-wide identification of epigenetically altered genes in HNSCC enhances an understanding of the mechanisms of carcinogenesis. Additionally, these approaches can uncover novel cancer-specific DNA methylation events that can be used for molecular detection strategies in surgical margins or bodily fluids [7, 24–27].

Recent data suggests that detection of promoter methylation of *EDNRB* and *DCC* in patients with high-risk oral lesions has a similar performance in diagnosis as expert clinical evaluation [25]. Nonetheless, tests utilizing *DCC* and *EDNRB* promoter methylation is limited to 46% sensitivity, and 72% specificity for cancer detection in salivary rinses [25]. While low sensitivity may be limited by the infrequency of cancer-related changes [11, 25, 28], low specificity raises technical concerns. Although increasing the test detection threshold can increase specificity [10, 25], this requires additional cut-off value manipulations that are not practical in the clinical setting. Therefore, detection of novel DNA markers with absolute specificity to cancer tissues can improve the current biomarker panels and enhance the potential clinical application of molecular detection strategies [7, 24–26]. Low sensitivity of individual biomarkers that are highly specific to cancer tissues can be overcome by combining several highly specific genes into the panels without sacrificing overall specificity [11].

Given the comprehensive nature of high throughput techniques, thousands of differentially methylated regions can be detected while comparing tumor and normal samples [10]. The heterogeneity of genetic and epigenetic alterations in solid tumors has presented challenges in using conventional statistical approaches, such as t-tests or signal-to-noise tests. These difficulties can be minimized by the use of outlier-based analyses to provide a measure of statistical significance for heterogeneous alterations in tumors. Outlier-based analysis has provided a mechanism to define significant, but diverse, alterations in cancers. The standard method employed in cancer research for outlier analysis is Cancer Outlier Profile Analysis (COPA [29]), which compares outliers to an empirical null. To eliminate low-signal outliers, this work implemented COPA-based statistics with a rank sum outlier approach as well as setting a minimum level for the calling of an outlier [30]. This is the first paper utilizing outlier analysis [30] for biomarker discovery.

## Materials and Methods

### Tissue samples

Primary tumor tissues and matched salivary rinse samples were collected from HNSCC patients at Johns Hopkins Hospital after informed, written consent was obtained. This study was approved by Johns Hopkins Medicine Internal Review Board (JHM IRB) and performed under research protocol NA_00036235. Overall, two independent cohorts of HNSCC patient specimens and normal control specimens were used. All human subjects that participated in this study were de-identified after the clinical data collection. The discovery cohort was composed of 44 primary HNSCC tissues and 25 normal mucosal samples from uvulopalatopharyngoplasty (UPPP) surgeries of non-cancer affected control patients from previously published studies [31–33]. The independent validation cohort included primary tumor tissues and matched salivary rinse samples from 59 HNSCC patients, 31 normal UPPP tissue samples, and 35 salivary rinse samples from non-cancerous patients. All primary tissue and bodily fluid specimens were stored at -140°C until use. All primary tissue samples were analyzed by investigators from the Pathology Department of Johns Hopkins Hospital (WHW and JAB). Tumor samples were confirmed to be HNSCC and were subsequently microdissected to yield at least 75% tumor purity. Clinical characteristics of the two cohorts are listed in S1 and S2 Tables. The distribution of HNSCC sub-types in both cohorts is representative of the distribution of head and neck cancer both in the United States and worldwide, including ~30% of HPV-related (HPV+) oropharyngeal SCC cases. The validity of this discovery cohort, and its suitability for multiple analyses has been demonstrated in multiple prior publications [31–34]. In an effort to reduce bias and obtain robust cancer unaffected controls, control patients were randomly selected from available UPPP tissue specimens and salivary rinses, but clinical characteristics were not able to be matched.

### DNA preparation

Microdissected tumor tissue samples or 250 μl aliquots of saliva samples were digested in 1% Sodium Dodecyl Sulfate (SDS) solution (Sigma) and 50 μg/ml proteinase K (Invitrogen) solution at 48°C for 48–72 hours. DNA was purified by phenol-chloroform extraction and ethanol precipitation as previously described [35]. DNA was resuspended in LoTE buffer, and DNA concentration was quantified using the NanoDrop spectrophotometer (Thermo Scientific).

### RNA preparation

RNA was isolated from the microdissected tissue samples with the mirVana miRNA Isolation Kit (Ambion) per manufacturer’s recommendations, and RNA concentration was quantified using the NanoDrop.

### Arrays

Ten micrograms of RNA and DNA were submitted to the Johns Hopkins Core Facility for quality control query and sample analysis by high-throughput arrays. Samples were run on Affymetrix HuEx1.0 GeneChips (containing 1.4 million probes) for expression analysis and Illumina Infinium HumanMethylation27 BeadChips (probing 27,578 CpG dinucleotides) for methylation analysis following bisulfite conversion. All arrays were run according to manufacturer protocols and the data was reported earlier [31–33]. Affymetrix Expression Data is available in GEO33205 and Illumina Methylation Data is available in GEO33202. Both data sets are available in GEO superSeries GSE33232. The data can be accessed at http://www.ncbi.nlm.nih.gov/geo/query/acc.cgi?acc=GSE33232.

### HPV analysis

Pathology reports regarding the HPV status of oropharyngeal SCC tumors were obtained from the Johns Hopkins Hospital Pathology Department. In addition, the HPV status of all oropharyngeal SCC primary tumor tissues was independently confirmed by quantitative PCR (qPCR) using HPV16 primers and probes on the real-time PCR machine [7] relative to CaSki (ATCC) cell line, known to have 600 copies of HPV16 per genome. Samples with HPV copy number ≥ 1 copy/genome/cell were identified as HPV positive.

### Bisulfite Treatment and Bisulfite Genomic Sequencing

The EpiTect Bisulfite Kit (Qiagen) was used to convert unmethylated cytosines in genomic DNA to uracil. Bisulfite-treated DNA was amplified with primers designed using MethPrimer [36, 37] (S3 Table). Primer pairs were designed within the CpG island surrounding the promoter region with close proximity to the methylation array probes. The representative sample from the discovery cohort were chosen on the basis of highest differences in methylation and concurrent expression as computed during outlier analysis for each individual gene. Bisulfite sequencing was chosen for this stage in order to ascertain the absolute (not relative or normalized) methylation status of several CpG dinucleotides in the CpG island near the promoter of each gene. Touch-down PCR was performed [38]. The PCR products were purified using the QIAquick 96 PCR Purification Kit (Qiagen) and purified PCR products were sequenced (Genewiz). Gene methylation status was determined as a trichotomous variable (unmethylated, hemimethylated, or hypermethylated) according to the sequence reads.

### Quantitative methylation-specific PCR (QMSP)

For QMSP, primers were designed to specifically include CpG dinucleotides that showed changes in methylation as seen by bisulfite sequencing. QMSP was performed on the real-time PCR machine with normalization to unmethylated *β-actin* reference control [39]. The Taqman PCR machine was set on 38 cycles maximum to eliminate false-positive signals. Bisulfite-converted leukocyte DNA from a healthy individual was used as a negative control. The relative level of methylated DNA in each sample was determined as a ratio of the amplified gene to *β-actin* [40] and multiplied by 100. Sequences of the primers and probes used can be found in S3 Table.

### Reverse Transcription and Quantitative Real Time PCR

One μg of RNA from the validation cohort was reverse transcribed using the High Capacity cDNA Reverse Transcription Kit. Quantitative real-time PCR was performed using gene-specific expression assays (S3 Table) and Universal PCR Master Mix on the 7900HT real-time PCR machine (all from Applied Biosystems) per manufacturer’s recommendations. Expression of the gene of interest was quantified in triplicates relative to *GAPDH* and *18S* expression using the 2-ΔΔCT method [41].

### Statistical analysis

#### Methylation data normalization

For the promoter methylation data, β values (proportion of methylation) were estimated from unmethylated (U) and methylated (M) measurements on a probe level basis. Gene level estimates were produced by choosing the highest methylation levels among all probes linked to the same gene (14,477 genes total).

#### Significant core probe determination

Gene expression data was normalized with robust multi-array average (RMA) analysis using the Bioconductor oligo package [42, 43]. Gene level estimates were produced by RMA using the core probes, yielding 22,011 genes for analysis [31, 32].

#### Outlier Analysis

The standard method employed in cancer research for outlier analysis is Cancer Outlier Profile Analysis (COPA), which compares the outlier distributions to an empirical null generated by permutation of class labels [29]. A modified rank sum outlier approach, modified from Ghosh [44], was used, in which minimum change levels were set for the definition of an outlier [30]. This eliminated many outliers in which change was not biologically meaningful (e.g., methylation change of less than 10% between any two samples). These statistics were applied to the discovery DNA methylation data set, containing 14,477 genes for 44 tumor tissues, in which signals from 25 normal samples were used to establish the base line cut-off point for each gene. Left-tail and right-tail outliers were determined using the rank-sum method [45]. Outlier scores were calculated for both right-tail and left-tail cases, which allowed the definition of outliers that were hypermethylated and hypomethylated on tumors, respectively [30, 45]. Given that outlier analysis does not have a score cut-off, a threshold was selected to yield approximately 50 top-scoring genes. This score cut-off of 13.2 identified 37 top candidates for further validation (S4 Table).

#### Expression-methylation correlation

Normalized gene expression data was correlated with promoter methylation levels in the methylation candidates using Spearman’s rank correlation (Table 1).

**Table 1:** Twenty four DNA methylation biomarker candidates.

#	Gene name	Gene description	Outlier score	Spearman's coefficient	p-value
1	*HHEX*	Transcription factor	67.51	-0.079	1.61E-05
2	*VILL*	Villin-like	47.65	-0.235	0.0103
3	*CHFR*	Checkpoint	37.24	-0.464	0.0010
4	*ZNF160*	Zinc finger	36.21	-0.440	0.0169
5	*FLJ22688 (FUZ)*	Fuzzy homolog	28.08	-0.290	0.0022
6	*RBP5*	Retinol binding	24.40	-0.123	0.0021
7	*MEF2C*	enhancer	23.48	-0.137	0.0080
8	*CLGN*	Chaperone protein	23.41	-0.135	0.0323
9	*IDUA*	iduronidase	21.79	-0.063	0.0635
10	*PIP5K1B*	kinase	20.68	-0.151	0.0003
11	*ADFP (PLIN2)*	Adipocyte differentiation	19.83	-0.220	0.0117
12	*ZNF420*	Zinc finger	19.46	-0.280	0.0125
13	*ZNF141*	Zinc finger	19.02	-0.355	0.0297
14	*ZNF211*	Zinc finger	17.78	-0.177	0.0118
15	*ENPP5*	phosphatase	16.47	-0.150	0.0023
16	*ZNF71*	Zinc finger	16.36	-0.058	0.0039
17	*CCND2*	Cyclin D2	16.26	-0.014	0.0036
18	*GLOXD1 (HPDL)*	Dioxygenase-like	16.19	-0.16	0.0002
19	*ICA1*	autoantigen	15.64	-0.327	0.0002
20	*ZNF14*	Zinc finger	15.45	-0.234	0.0208
21	*HAAO*	dioxygenase	15.41	-0.072	0.0635
22	*RECK*	MMP9 regulator	15.04	-0.222	0.0054
23	*ITPKB*	Inositol kinase	14.61	-0.090	0.0089
24	*ZNF585B*	Zinc finger	14.41	-0.396	0.0017

The genes are ranked by outlier score. Spearman's coefficient was calculated to evaluate DNA methylation and gene expression correlation. P-values for testing the DNA methylation differences between tumor and normal groups were calculated using t-test

#### Correlation and Concordance of DNA Methylation Detection

Correlations of ZNF DNA methylation signals between primary tumor tissues and salivary rinses (detected by QMSP assay) among HNSCC patients from the validation cohort were evaluated via Spearman’s coefficient and kappa coefficient. Agreement concordance was calculated using kappa statistics.

#### Sensitivity and Specificity Quantitation

The QMSP methylation values for saliva samples were calculated using a standard curve method and were normalized to a methylation-independent DNA loading control (*β-actin*). Methylation level of each gene was treated as a binary variable (methylated vs. unmethylated) by dichotomizing the methylation at zero DNA methylation detection by QMSP. Subjects with a diagnosis of HNSCC were defined as “presence of disease”, and the “absence of disease” subjects were defined by normal controls. True positive, true negative, false positive and false negative rates were then determined for the individual genes. For the combination of markers, the patient was classified as “test positive” if any of the markers were positive, and “test negative” if all the markers were negative. Sensitivity was estimated as the proportion of patients who were test positive among those with disease, and specificity was estimated as the proportion of patients who were test negative among those without disease. The 95% Confidence Interval (CI) for sensitivity and specificity was calculated assuming binomial distribution [46].

## Results

### Identification of differentially methylated gene outliers

**Fig 1:**
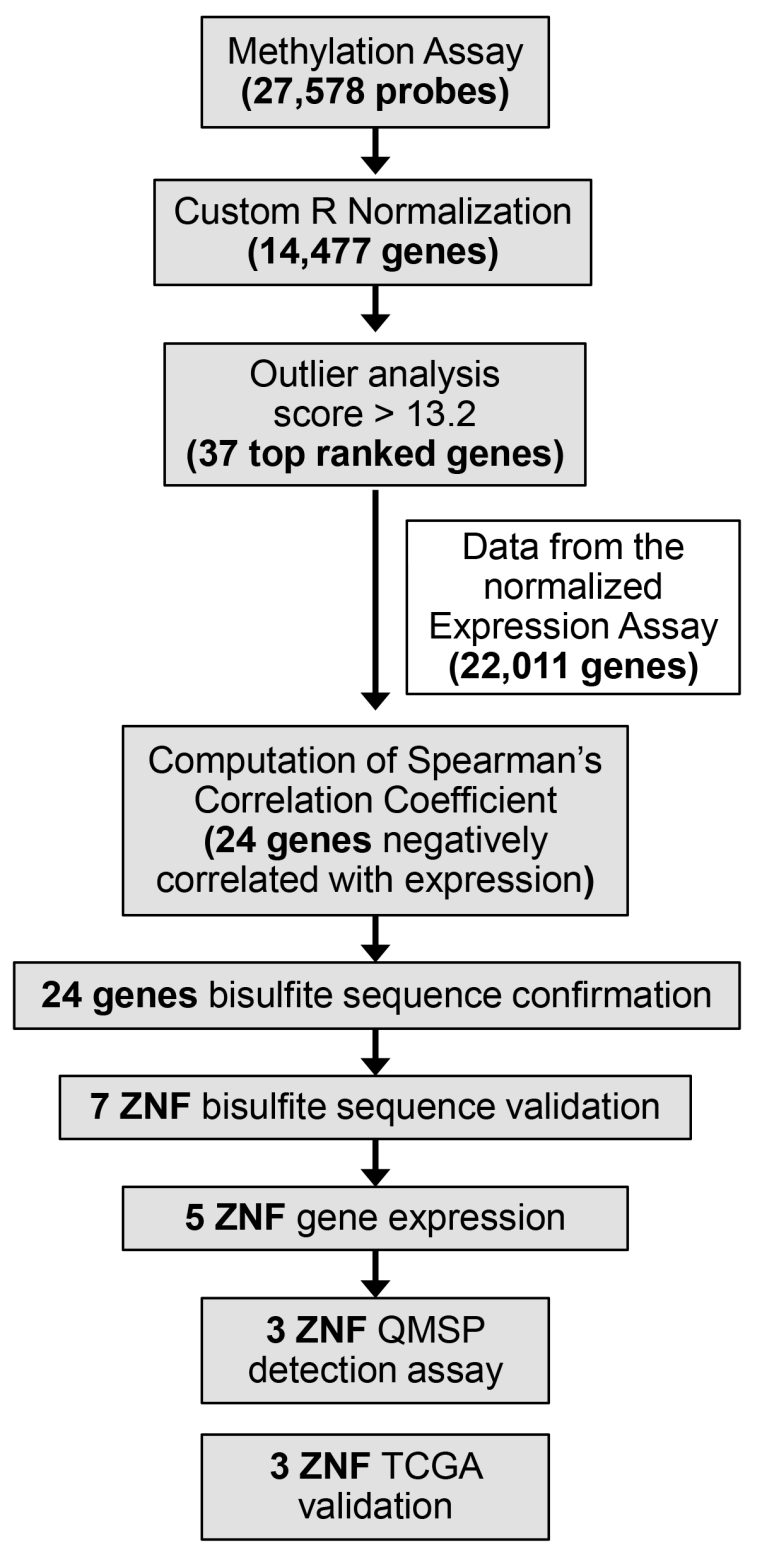
Integrative methylation screening strategy. Schematic outline of the integrative approach utilized in this study, which combines high-throughput screening of DNA methylation and gene expression for the discovery cohort of HNSCC: employment of DNA methylation array data with 27,578 probes total; normalization of the data in R, 14,477 genes total; outlier analysis and cut-off to receive approximately 50 top genes (13.2 outlier score; 37 top ranked genes passed, see Methods for details); Integration of the normalized data from the expression assay (22,011 genes); Spearman’s correlation coefficient calculations (24 genes passed); 7 ZNFs bisulfite sequencing validation; qRT-PCR, 5 ZNFs gene expression validation; Validation of 3 ZNF QMSP detection in saliva and tumor samples in different cohorts.

From genome-wide differential DNA methylation analysis of 44 primary tumors and 25 normal tissue controls, biomarker candidates were selected based on the scheme shown in Fig 1. Based on the number of outlier samples and the relative signal intensity, 37 of the top ranking candidates were chosen for further analysis (S4 Table). Correlation of the expression and methylation array data allowed for the discovery of 24 candidate genes (out of 37 initial genes) with biologically relevant negative correlation between DNA methylation and gene expression (Table 1). Notably, all 24 candidate genes showed hypermethylation and decreased expression in tumor samples. Furthermore, all candidates showed minimal DNA methylation signal in normal tissues, with maximal mean of β-value for normal samples of 0.058, or 6% methylation (S1 Fig and S5 Table). The standard t-test demonstrated that 23 out of 24 genes (96%) had statistically significant difference in DNA methylation between normal and all tumor samples and between normal and HPV- tumor samples. One gene, *CCND2*, did not reach statistical significance based on a t-test, however this did demonstrate a difference between tumor and normal samples by a Fisher Exact test based on presence of hypermethylated outliers in tumor samples.

### Increased methylation and decreased gene expression alterations in the HPV- patients

It is known that HPV+ and HPV- HNSCC cases differ in their landscape of genetic and epigenetic alterations [5, 12, 47, 48]. By separating 44 HNSCC patients by HPV status, it was determined that 92% (22 of 24) genes had significantly higher methylation in HPV- HNSCC, as compared to normal samples (S5 Table). Only 4% (1 of 24) candidates had DNA methylation in HPV+ HNSCC samples significantly higher relative to normal samples (S5 Table). The majority, 54% (13 of 24) genes had significantly higher methylation in HPV- HNSCC, as compared to HPV+ samples, in agreement with published data [12]. The tumor samples had a corresponding decrease of candidate gene expression (S1 Fig and S6 Table). Thus, 71% (17 of 24) genes demonstrated a significant decrease in gene expression in all HNSCC samples compared to normal samples; 75% (18 of 24) genes showed a significant decrease in gene expression in HPV- samples compared to normal samples; and 21% (5 of 24) genes had significantly decreased gene expression in HPV- samples compared to HPV+ samples. The decreased gene expression in tumor samples was consistent with the overall increased promoter methylation in tumor samples (S1 Fig and Table 1).

### Validation of promoter hypermethylation of candidate genes

**Fig 2:**
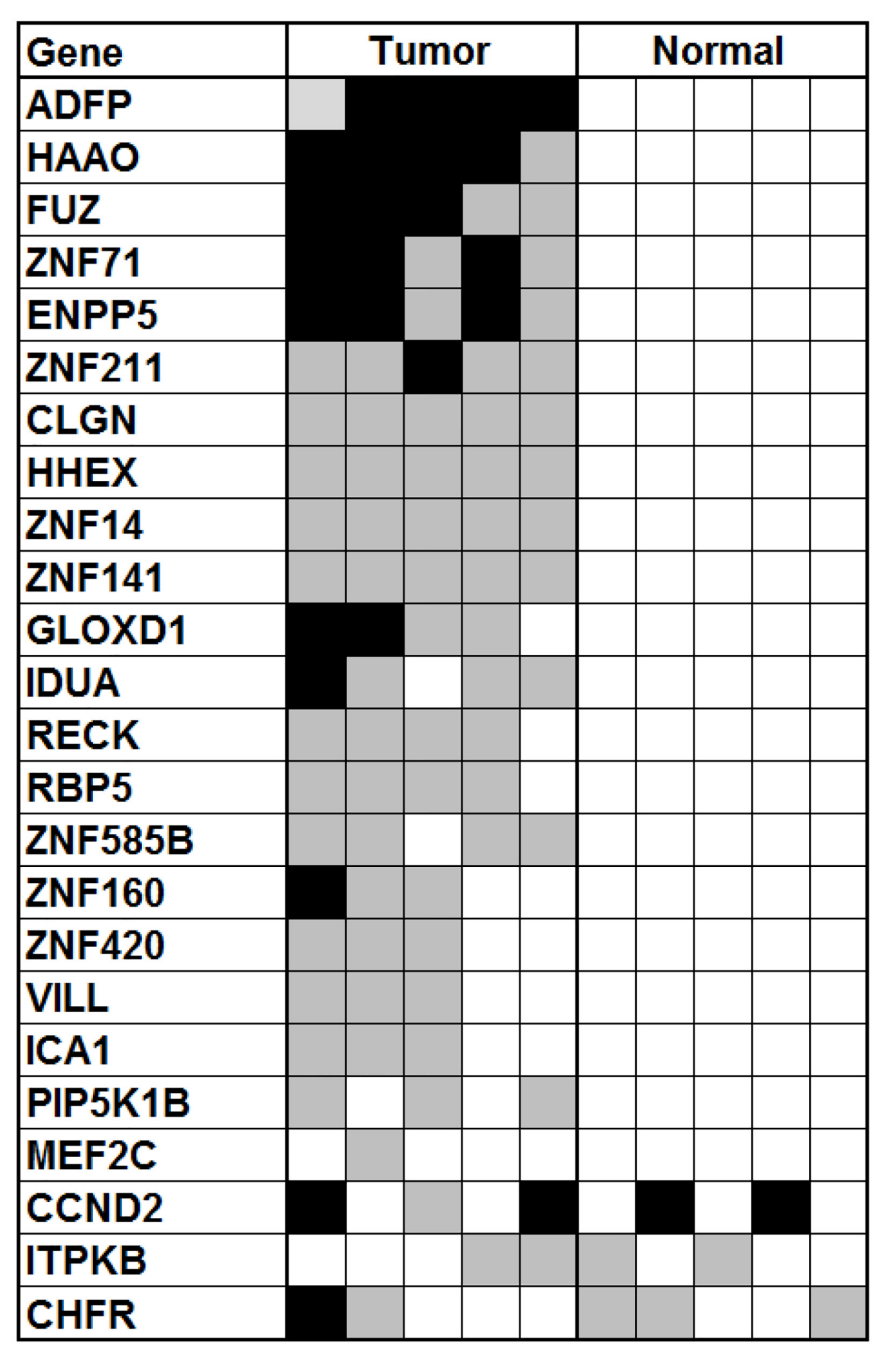
Promoter DNA hypermethylation of prospective tumor suppressor genes. Bisulfite sequencing results are shown in 5 HNSCC tumor samples and 5 normal tissues from the original discovery cohort for the 24 top-scoring candidate genes (Table 1). Shaded black boxes represent completely methylated promoters, gray boxes represent hemimethylated promoters, and white boxes represent unmethylated promoters.

To validate the differential methylation status of the CpG islands near the promoter region of the 24 selected candidate genes, bisulfite sequencing was performed on 5 representative samples of normal mucosal samples and 5 primary HNSCC tumor samples from the initial discovery cohort. Of the 24 genes, 22 (92%) showed increased methylation in tumors relative to normal samples (Fig 2). Twenty candidate genes (84%) showed methylation in greater than 50% of primary tumor, including *ADFP*, *FUZ*, *ZNF71*, *ENPP5*, *ZNF211*, *and ZNF14* (Fig 2). Bisulfite sequencing data strongly correlated with the results from array data, in which minimal DNA methylation was detected in normal samples (S1 Fig and Fig 2).

### Zinc Finger Protein candidates

It was noted that 7 out of 24 candidate genes were members of the Zinc Finger Protein (ZNF) group, and additional analysis was performed in this group. In order to elaborate on ZNF candidates, DNA methylation was analyzed in a separate validation cohort of 59 tumors and 31 normal tissues (S2 Table). Using bisulfite sequencing methods, significant increase of DNA methylation for five of the genes was detected in this cohort (S7 Table). However, decreased DNA methylation of *ZNF141* promoter in the validation cohort contradicted the discovery cohort data, and, no DNA methylation was detected in *ZNF211* in samples from the validation cohort. Of all remaining five ZNF protein genes, *ZNF14*, *ZNF160*, *ZNF71*, *ZNF420* and *ZNF585B*, DNA methylation was significantly higher in tumor samples, as compared to normal controls (S7 Table).

### ZNF downregulation is associated with promoter methylation

To validate the hypothesis that expression of individual ZNF protein genes is affected by methylation of their promoters, qRT-PCR analysis was subsequently performed for *ZNF14*, *ZNF71*, *ZNF160*, *ZNF420* and *ZNF585B* expression on the samples from an independent validation cohort (S2 Fig). All but *ZNF71* demonstrated significant downregulation of expression in tumor samples compared to normal tissues, which was consistent with increased methylation levels. Separation of tumor samples according to HPV tumor status demonstrated that ZNF expression was not significantly different in HPV- and HPV+ patient groups (S2 Fig and S7 Table).

### ZNF DNA methylation detection is highly specific for primary HNSCC tissues

Based on the DNA methylation results, it was hypothesized that ZNF methylation could potentially be used as a clinically applicable biomarker for HNSCC. Thus, QMSP primers and probe assays were designed for *ZNF14*, *ZNF160* and *ZNF420*. A highly specific QMSP assay for *ZNF585B* could not be designed due to its high CpG density.

**Fig 3:**
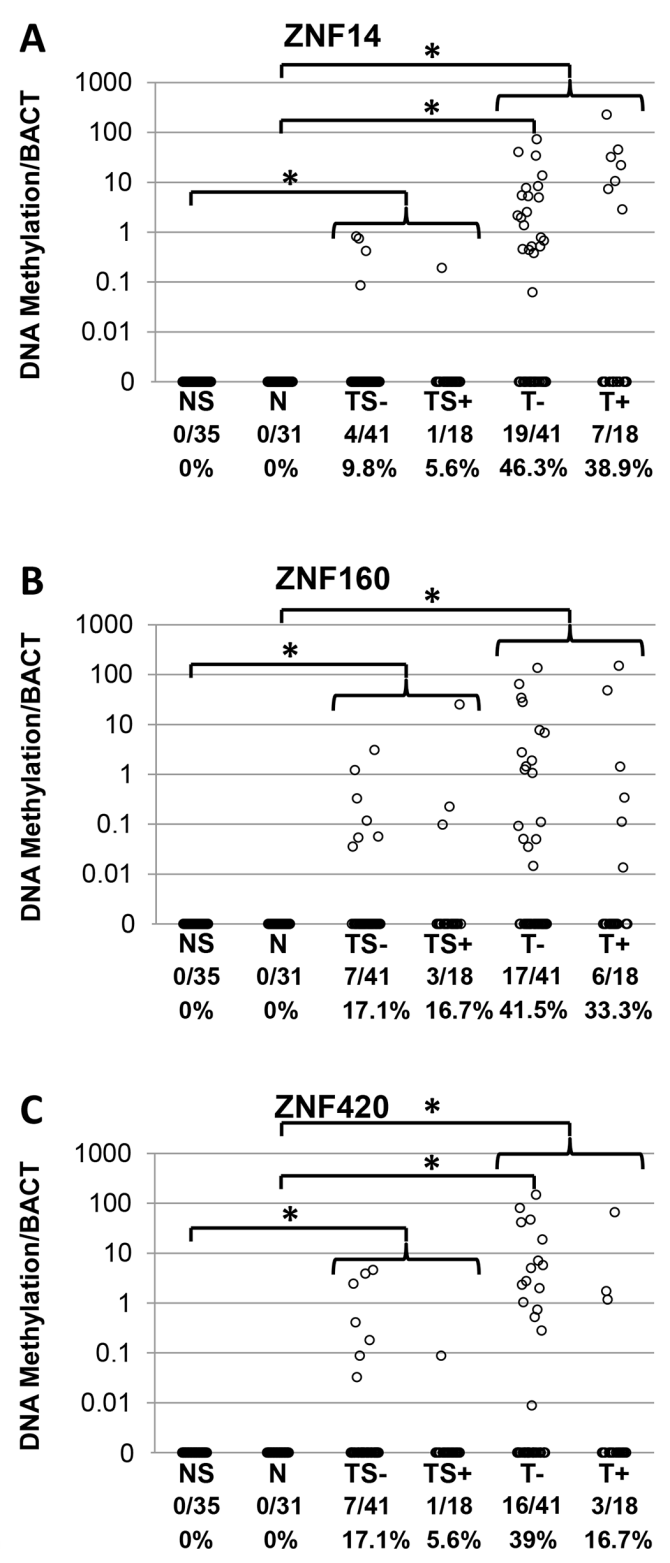
*ZNF14*, *ZNF160*, and *ZNF420* DNA methylation detection in primary tissues and bodily fluids of HNSCC patients. Shown are ZNF QMSP results in 59 HNSCC primary tumor and salivary rinse samples compared to normal plasma and salivary rinse samples from the validation cohort. ZNF promoter methylation was quantified relative to BACT methylation and multiplied by 100. Plus (+) or minus (-) refers to their HPV status of the cancer patients. NS = normal salivary rinse sample (n = 35), N = normal primary tissues (n = 31), TS = salivary rinse from HNSCC patients (n = 59, 41 of HPV+ [TS+] and 18 of HPV- [TS-]), T = primary tumor samples from HNSCC patients (n = 59). There was no detectable ZNF DNA methylation in normal samples. Significant (p<0.05) difference between groups is indicated by an asterisk (*), as calculated by the Fisher exact test.

The QMSP assays were first validated for all three ZNF genes in tissue samples. Similar to bisulfite sequencing results, QMSP assays did not detect DNA methylation in any normal samples, but DNA methylation was detected in *ZNF14*, *ZNF160* and *ZND420* in 44.1%, 39% and 32.2% of tumors respectively. At least one ZNF was methylated in 57.6% of tumor tissues. (Table 2). Notably, 17% of patients (10 of 59) had methylation detected on all three ZNF promoters. Slightly higher rates of DNA methylation were seen in the HPV- group, but the difference was not statistically significant (Fig 3).

**Table 2:** Correlation, concordance and agreement of ZNF DNA methylation signal in salivary rinses and in primary tissues from the validation cohort.

	Methylated DNA in saliva (n = 59)	Methylated DNA in tissue (n = 59)	Methylation level correlation	Concordance	Agreement
	n (%)	n (%)	Spearman correlation (p-value)	Kappa coefficient (95% CI)	%
*ZNF14*	5 (8.5)	26 (44.1)	0.1 (0.4519)	0.14 (-0.03, 0.3)	**61.02**
*ZNF160*	10 (16.9)	23 (39)	0.25 (0.0565)	0.25 (0.02, 0.47)	**67.8**
*ZNF420*	8 (13.6)	19 (32.2)	**0.55 (<0.0001)**	**0.5 (0.26, 0.73)**	**81.36**
Any ZNF	13 (22)	34 (57.6)	NA	0.28 (0.11, 0.46)	**61.02**

Significant values are in bold.

### ZNF DNA methylation detection in TCGA cohort

To validate ZNF DNA methylation and its correlation with expression in different HNSCC cohorts, the Illumina Infinium HumanMethylation450 BeadChip array and RNA-Seq data were downloaded and analyzed from The Cancer Genome Atlas (TCGA) HNSCC cohort, which contains 279 HNSCC tumors, including 36 HPV+, with matched normal samples. ZNF DNA methylation was minimal in TCGA normal samples (S3 Fig and S10 Table), in agreement with the discovery and validation cohort data. All three ZNFs had strong negative correlation of DNA methylation and expression, especially *ZNF420* (S3 Fig). Notably, out of all DNA methylation probes available from TCGA, probes within *ZNF420* promoter had the highest rate of DNA methylation in HNSCC samples with minimal methylation detection in normal samples. Overall, we observed a high concordance between the discovery, validation and TCGA cohorts for the detection of ZNF promoter methylation using four different techniques for DNA methylation detection (S9 Table).

### ZNF DNA methylation detection as prospective HNSCC biomarkers

To test the performance of developed QMSP assays for ZNFs for the detection of DNA methylation in bodily fluids, matched salivary rinse samples from the HNSCC validation cohort patients were used. Comparison of DNA methylation signals in the salivary rinse samples of HNSCC and non-cancerous patients demonstrated that the specificity of the combined ZNF panel to detect HNSCC in these bodily fluids was 100% (95% CI: 89.9% - 100%), and the sensitivity was 22% (95% CI: 12.3% - 34.73%) (Table 3). Notably, the exploration of ZNF methylation correlation with clinical data by different statistical analyses did not show any significant associations.

**Table 3:** Promoter DNA hypermethylation detection in salivary rinses of HNSCC and non-cancerous patients from the validation cohort.

	HNSCC (n = 59)	Control (n = 35)	Sensitivity	Specificity	Fisher's exact test
	n	n	% (95% CI)	% (95% CI)	p-value
*ZNF14*	5	0	8.47 (2.81–18.68)	100 (89.9–100)	0.1534
*ZNF160*	10	0	16.95 (8.44–28.97)	100 (89.9–100)	0.0119
*ZNF420*	8	0	13.56 (6.04–24.98)	100 (89.9–100)	0.0237
Any ZNF	13	0	22.03 (12.3–34.73)	100 (89.9–100)	0.0016

## Discussion

Despite the development of high throughput technologies and the identification of promising DNA methylation markers, no one HNSCC biomarker has currently been accepted for clinical detection and surveillance. A key concern in recently proposed HNSCC biomarkers is the high false-positive rate. As HNSCC is a highly heterogeneous disease, it is challenging to define a single biomarker with both high sensitivity and specificity [10, 11, 25]. Many proposed biomarkers have reasonable sensitivity but relatively low specificity, and combinations of these biomarkers would likely lead to increased rate of false-positives [25]. Use of conventional statistical methods may underestimate heterogeneous alterations in malignancy; however, these challenges can be overcome using recently developed outlier analysis adapted from COPA. To our knowledge, this is the first published work that utilizes outlier analysis for DNA methylation biomarker development. While the ZNF group of candidates studied in this work provided 100% specificity, additional studies may boost sensitivity by validating more methylated gene candidates for the development of a multi-gene DNA methylation-based panel of HNSCC biomarkers.

There are a number of publications that utilize high throughput DNA methylation analysis, however many have limited cohort sizes [11–13]. This study was able to use a previously published cohort consisting of 44 tumor and 25 normal controls for the discovery of potential methylated biomarkers. Furthermore, biomarkers were additionally validated in a larger independent cohort (with 59 tumor samples) and in TCGA (279 tumor samples). The use of the Illumina 27 DNA methylation array, which has been used successfully by others [10–14], allowed for the definition of highly specific prospective biomarkers for HNSCC. However, it is acknowledged that more comprehensive DNA methylation platforms may enable the discovery of larger numbers of candidate alterations.

Correlation of methylation and expression is another powerful tool that was utilized in this study to identify biologically relevant methylation changes that likely occur earlier in cancer development [10, 14]. Only methylation changes that occur earlier in tumor development will allow for development of subsequent gene expression changes. DNA methylation changes that did not correlate with gene expression were eliminated to define a more focused panel of 24 candidate methylated gene biomarkers. Furthermore, correlation of DNA methylation with expression from the available gene expression array helps to reduce single platform bias. Multiple validation steps were performed in this study which included a total of three cohorts of HNSCC samples, as well as four different detection techniques for DNA methylation (Illumina arrays 27 and 450, bisulfite sequencing and QMSP) and three different techniques for gene expression (Affymetrix array, RNA-Seq from TCGA and qRT-PCR). Consistent results were observed between different cohorts and techniques (S9 Table). These validation steps confirmed high specificity and reliability of the detected ZNF DNA methylation-based biomarkers for HNSCC.

Higher levels of promoter methylation were observed in HPV- patients in several putative tumor suppressor genes including Zinc Finger family members, consistent with previously published reports [12–14]. Significant DNA promoter hypermethylation and decreased gene expression of *ZNF420* were found in HPV- HNSCC patient groups when compared to HPV+ patients from both discovery and TCGA cohorts (S5 and S6 and S10 and S11 Tables). Differences between HPV+ and HPV- groups were not observed in *ZNF14* and *ZNF160*, however, these observations may have been limited by small HPV+ sample size.

Of the 24 biomarker candidates confirmed through expression correlation, seven (29%) belong to the Zinc Finger protein group, whose members have previously been shown possess tumor suppressor activity [13]. Additionally, all three prospective biomarkers (*ZNF14*, *ZNF160* and *ZNF420*) have a KRAB-A domain, and *ZNF160* and *ZNF420* are found on chromosome 19. Indeed, there are over 800 ZNF genes containing over 8000 ZNF domains in the human genome [49, 50]. Interestingly, the majority of ZNF gens (266 genes) are found on chromosome 19, where 202 of them are KRAB- ZNF genes [49]. The KRAB box-A is a transcription repression module, which supports the hypothesis that the proposed ZNF candidates are prospective tumor suppressor genes. All but *ZNF14* are located on the 19q13 locus, which was shown to be epigenetically silenced in oropharyngeal cancer [13, 14], supporting the hypothesis that *ZNF160*, *ZNF420*, *ZNF585B*, and *ZNF71* are epigenetically regulated in a coordinated fashion. Consistent with this model, the three prospective ZNF biomarkers were found to be hypermethylated in tumor cells with a corresponding decrease in gene expression in both this study and in the literature [13].

Salivary rinse biomarkers hold promise as inexpensive and non-invasive diagnostic tools particularly for head and neck malignancies, and have shown sensitivity and specificity comparable to physical examination in selected clinical settings [25]. In this study, the performance of ZNF DNA methylation detection was evaluated in both HNSCC primary tissues and salivary rinses. High correlation and high specificity of ZNF candidates was observed for tissue and saliva, showing that detection of ZNF DNA methylation has potential for use in a non-invasive HNSCC diagnosis assay. The low sensitivity of these biomarkers would require enhancement by combining these ZNF biomarkers with additional candidate biomarkers either this work or other similar studies [10, 11, 25]. This combination of highly specific biomarkers has the potential to lead to the development of a powerful, clinically appropriate diagnostic tool.

There are several limitations of this study that warrant discussion. First, clinical characteristics between normal and tumor patients were not able to be matched, and these clinical differences may potentially contribute to methylation changes. However, a robust and steady distribution of DNA methylation signals for all control samples, regardless of clinical characteristics has been observed in these cohorts, as previously published [31–34]. Moreover, these analyses were able to be confirmed on the TCGA cohort of matched tumor-normal populations. Second, the absence of reliable commercial ZNF antibodies for *ZNF14*, *ZNF160* and *ZNF420* limited our ability to confirm the protein level of gene expression during validation steps. However, expression data were robust enough to confirm the downregulation of candidate gene expression in different cohorts. Furthermore, the proposed panel of three ZNF genes had limited sensitivity in saliva samples. However, our results are comparable to the most prominent methylation-based HNSCC biomarkers currently available for detection of HNSCC-specific DNA in saliva: *EDNRB* (78% specificity and 38% sensitivity) and *DCC* (88% specificity and 27% sensitivity) [25]. Despite the lower sensitivity of the ZNF genes relative to *EDNRB* and *DCC*, the 100% specificity of the individual ZNF genes suggests that ZNFs are promising candidates for HNSCC detection in a combination with *ENDRB*, *DCC*, or even with other candidates from Table 1 and S1 Fig, such as *HHEX* or *FUZ/FLJ22688*. In this prospective combined panel of biomarkers, the exclusive specificity of individual biomarkers towards cancer samples will increase the sensitivity of the entire gene panel without sacrificing specificity. In conclusion, combining multiple high throughput platforms (methylation and gene expression), use of outlier statistics, and vigorous validation allowed for robust identification of novel biomarkers for the detection of HNSCC. In addition, ZNF genes were highly represented in top scoring biomarker candidates and showed consistent hypermethylation in tumors with corresponding down regulation of gene expression. Furthermore, DNA methylation changes in selected ZNF genes (*ZNF14*, *ZNF 160*, *and ZNF420*) are detectable in both tumor and saliva of HNSCC patients with high specificity and represent promising biomarkers for the development of non-invasive assays for the detection and surveillance of HNSCC. Nonetheless, for these biomarker to be translated to the clinic, further intense pre-clinical evaluation will be required to define and validate the performance of these biomarkers in the clinical setting.

## Supporting Information

S1 Fig Boxplots for the methylation (β-values, left) and expression (logarithm values of expression intensity, right) values for the 24 candidate genes from the discovery cohort. B-values and expression intensity values were from Illumina Infinium HumanMethylation27 BeadChips and Affymetrix HuEx1.0 GeneChips arrays, respectively. HNSCC population was separated according to their HPV status to HPV- and HPV+ samples. The length of each box is the inter-quartile range and represents the middle 50% of the values. The horizontal line inside the box depicts the median. The lower and upper hinges of the box represent the 25th and 75th percentiles, respectively. The vertical dashed lines extend from the box to the upper and lower 1.5 inter-quartile values from the upper and lower hinges. The empty circles represent the outliers above and below the upper and lower hinges. The t-test p-values comparing group of patients for these 24 genes can be found in S5 and S6 Tables for methylation and expression values, respectively.
(PDF)

S2 Fig Expression of ZNFs in the validation cohort. ZNF gene expression is shown relative to GAPDH. The boxplots were built as described for S1 Fig. Statistical analysis of the data can be found in S8 Table.
(PDF)

S3 Fig Boxplots for the methylation (β-values, Illumina Infinium HumanMethylation450 BeadChips, left) and expression (logarithm values of expression intensity, RNA-Seq, right) of *ZNF14* (top), *ZNF160* (middle), and *ZNF420* (bottom) genes in TCGA cohort. The boxplots were prepared as in S1 Fig. The t-test p-values comparing groups of patients for these 24 genes can be found in S10 and S11 Tables for methylation and expression values, respectively.
(PDF)

S1 Table Clinical characteristics of HNSCC patients from the discovery cohort. (PDF)

S2 Table Clinical characteristics of HNSCC patients from the validation cohort. (PDF)

S3 Table Primers, probes, and expression assays used in the study. (PDF)

S4 Table Outlier scores for 37 candidates from the methylation arrays. (PDF)

S5 Table DNA methylation β-values in different patient groups in the discovery cohort (Illumina Methylation array 27). These groups were compared by t-test.
(PDF)

S6 Table Gene expression values in different patient groups in the discovery cohort (Affymetrix Exon array). These groups were compared by t-test.
(PDF)

S7 Table Bisulfite sequencing of the promoter DNA hypermethylation detection in primary tissues from HNSCC and non-cancerous patients of the validation cohort. (PDF)

S8 Table QRT-PCR gene expression values in different patient groups in the validation cohort. These groups were compared by t-test.
(PDF)

S9 Table Comparison of the promoter DNA hypermethylation detection in all primary tissues from HNSCC and non-cancerous patients from three cohorts using four different DNA methylation detection techniques. (PDF)

S10 Table DNA methylation β-values in different patient groups in the TCGA-HNSCC cohort (RNA-Seq). These groups were compared by t-test.
(PDF)

S11 Table Gene expression values in different patient groups in the TCGA-HNSCC cohort (RNA-Seq). These groups were compared by t-test.
(PDF)
