# Supplementary figures and images for: Outlier Analysis Defines Zinc Finger Gene Family DNA Methylation in Tumors and Saliva of Head and Neck Cancer Patients
Source: PLoS One. 2015 Nov 6;10(11):e0142148. doi: 10.1371/journal.pone.0142148 (PMC4636259; doi:10.1371/journal.pone.0142148)

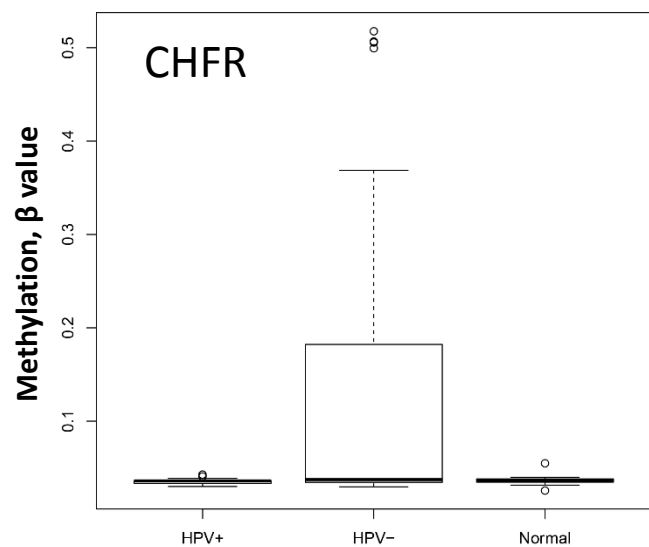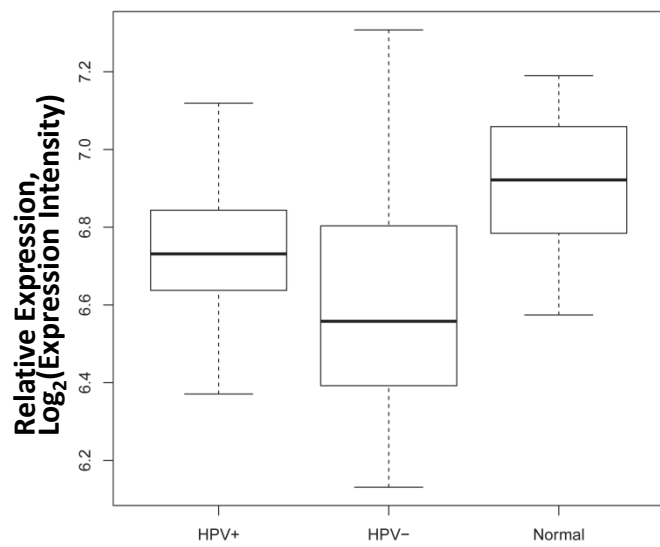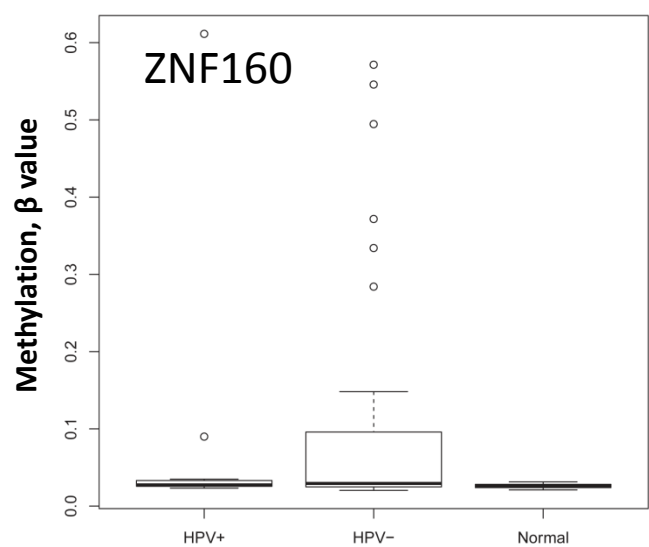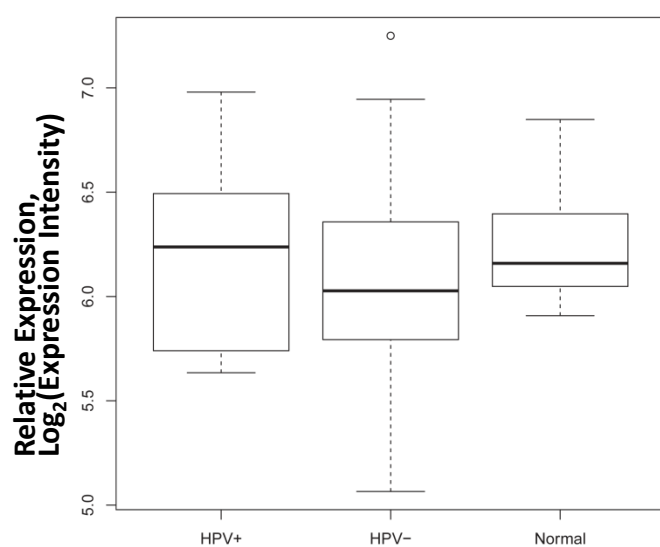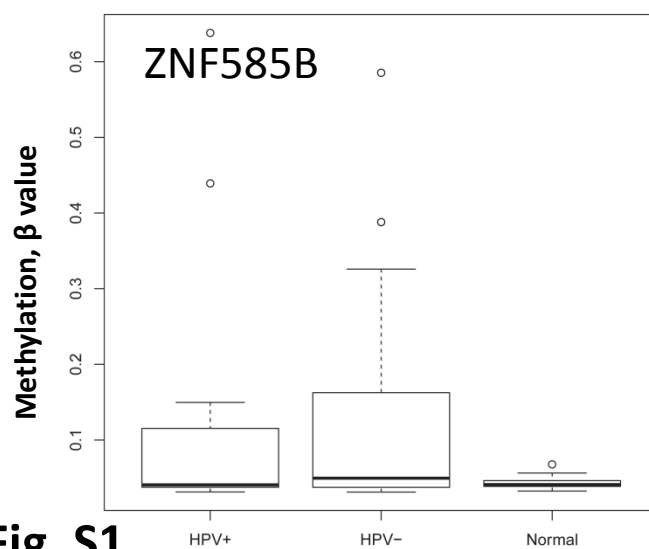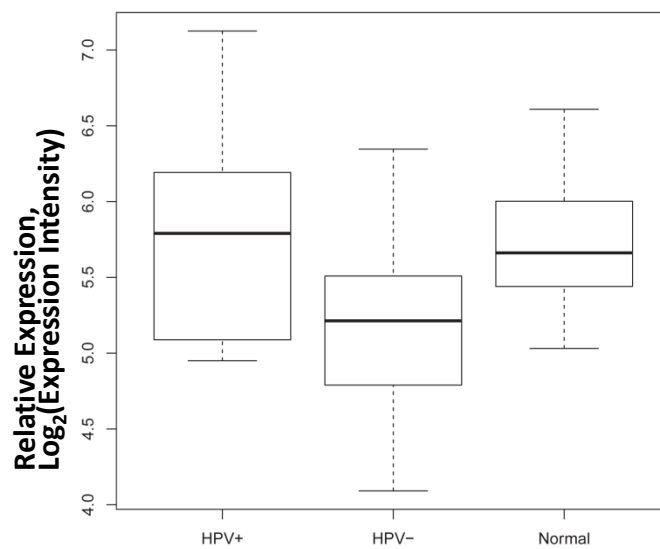

**Fig. S1**

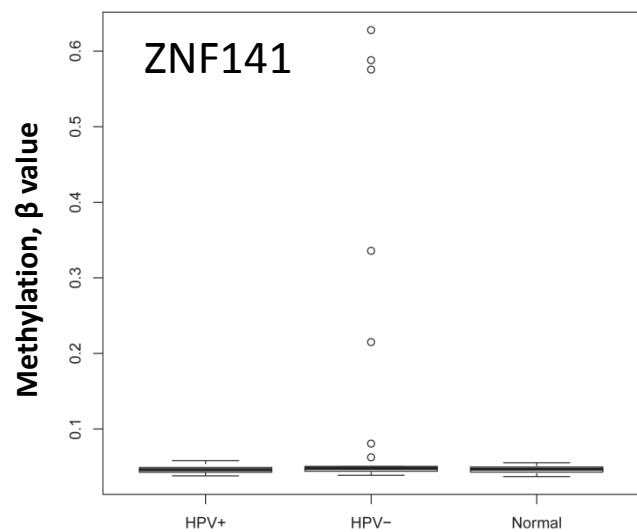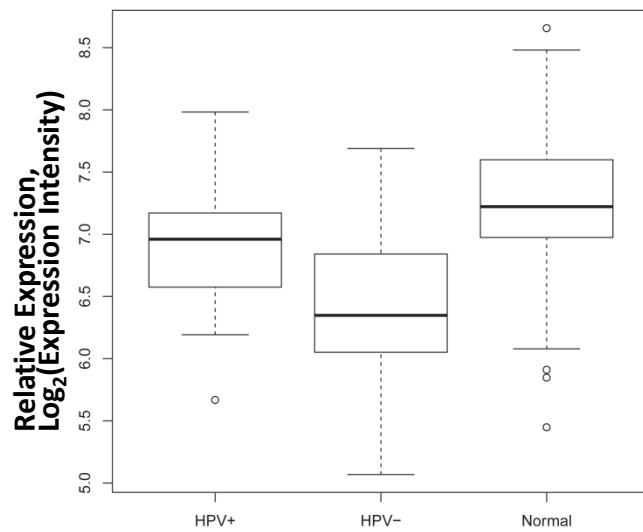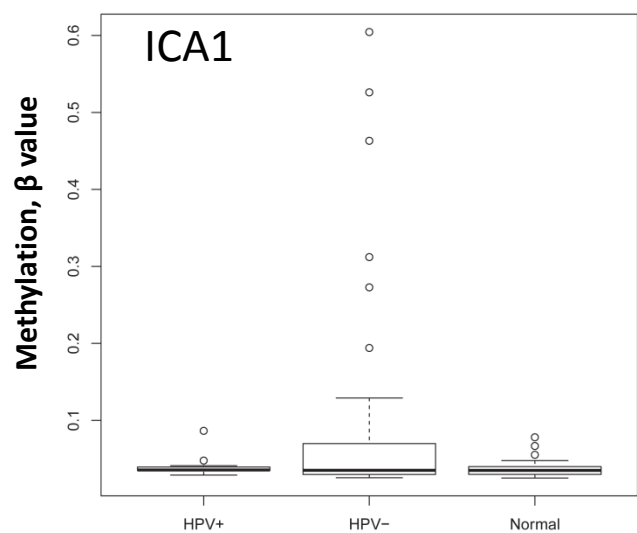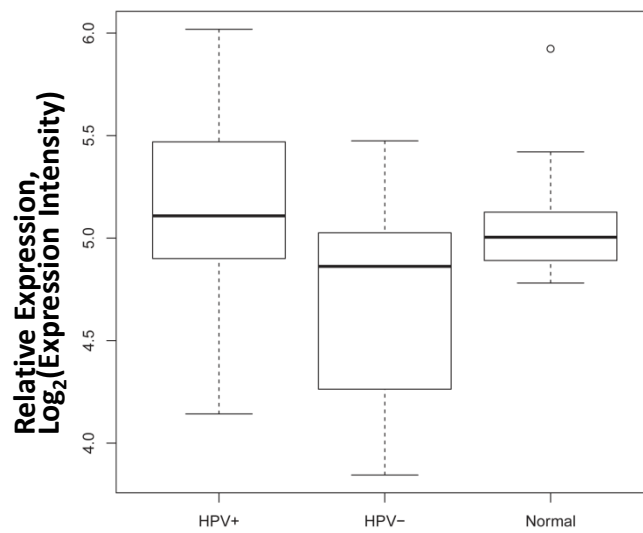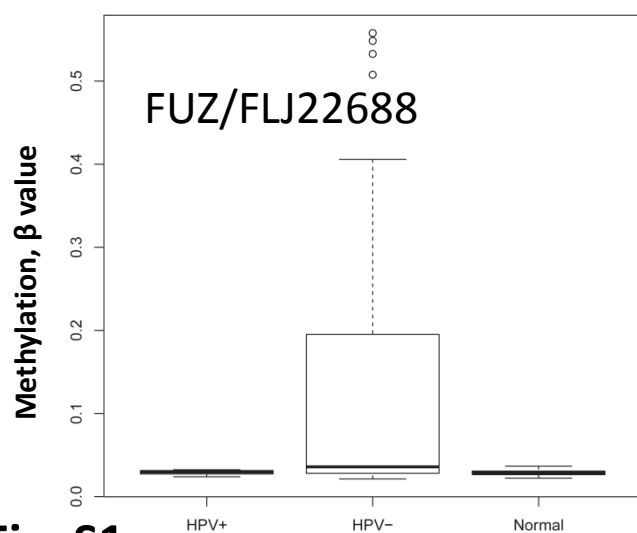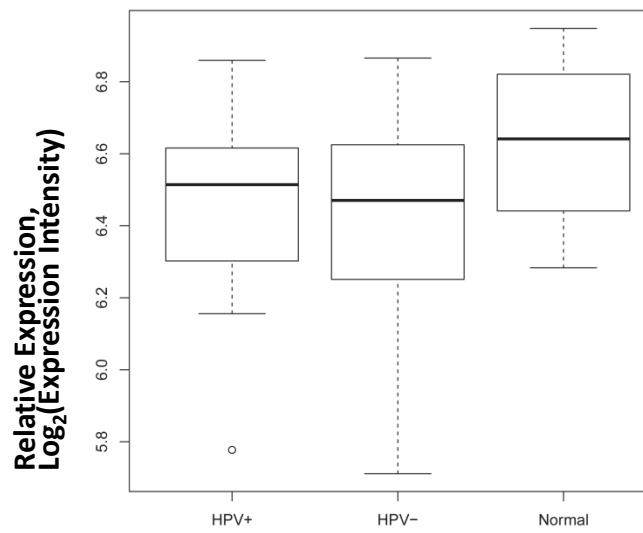

**Fig. S1**

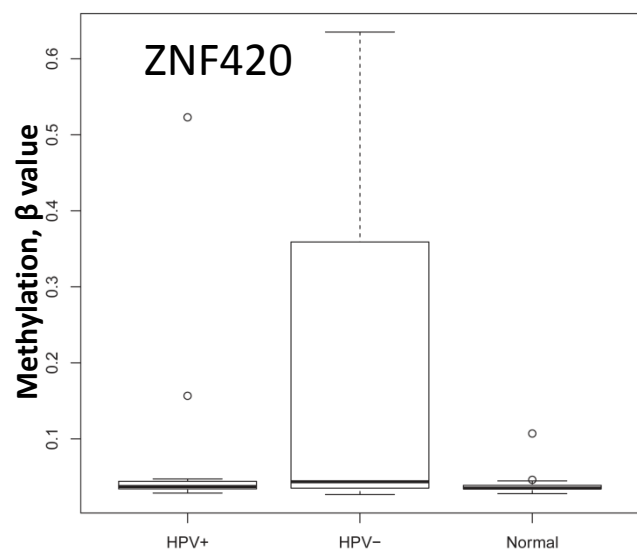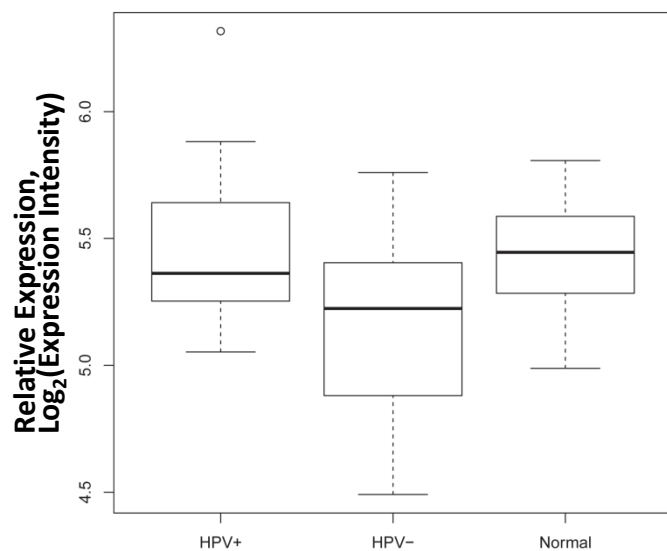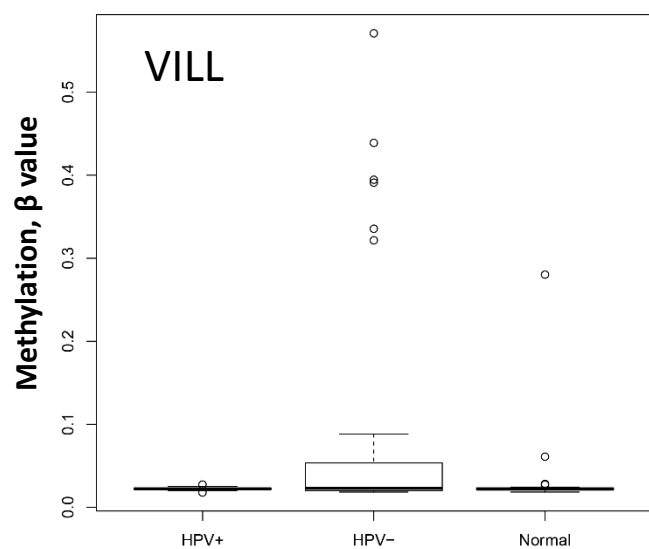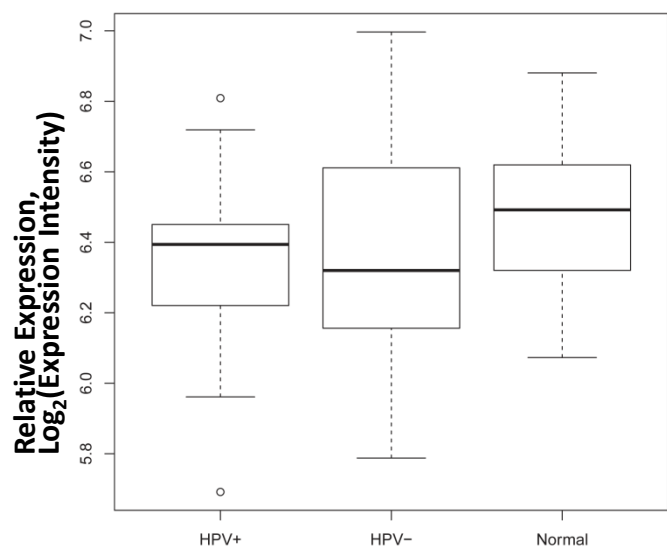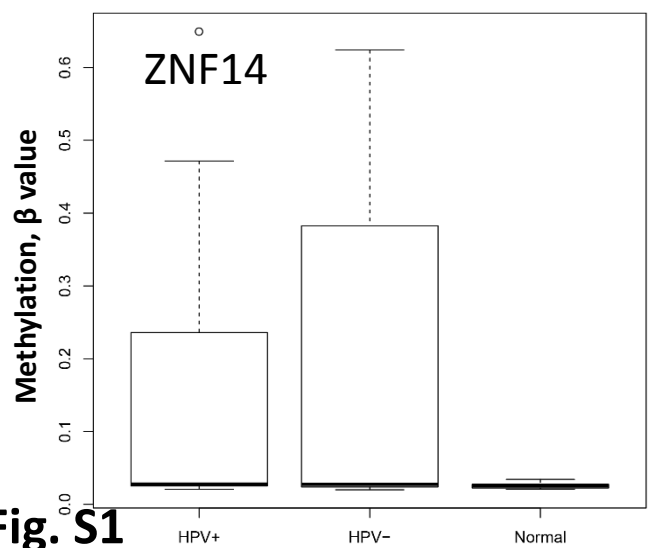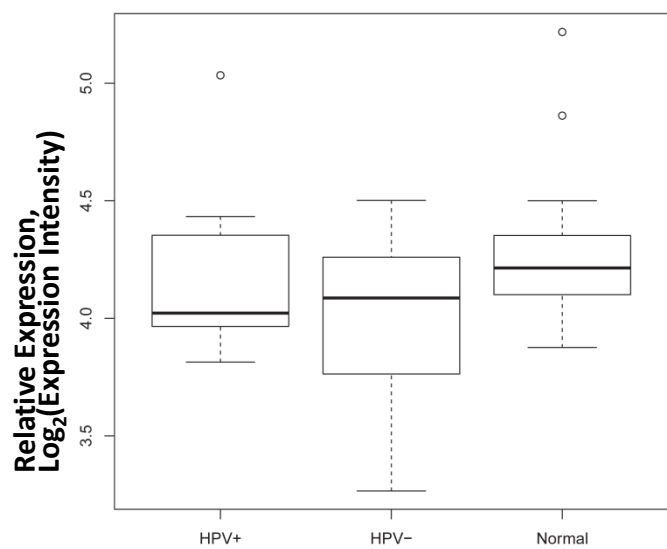

**Fig. S1**

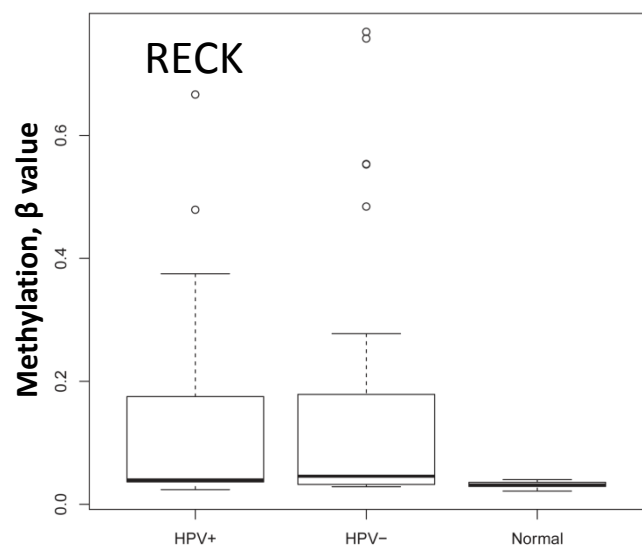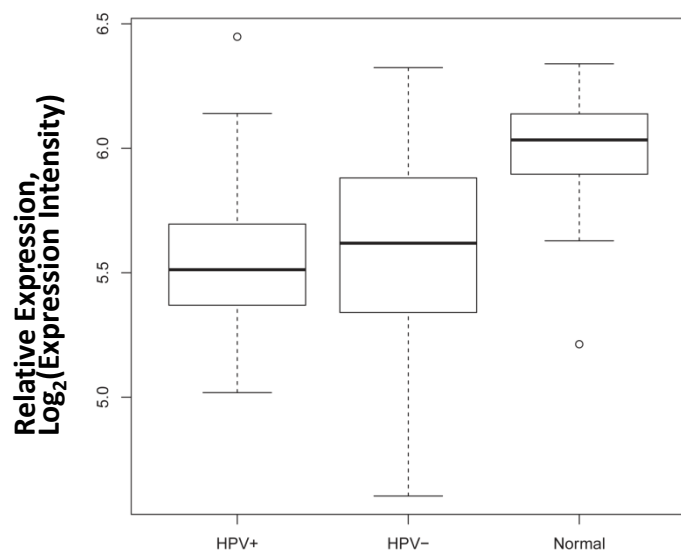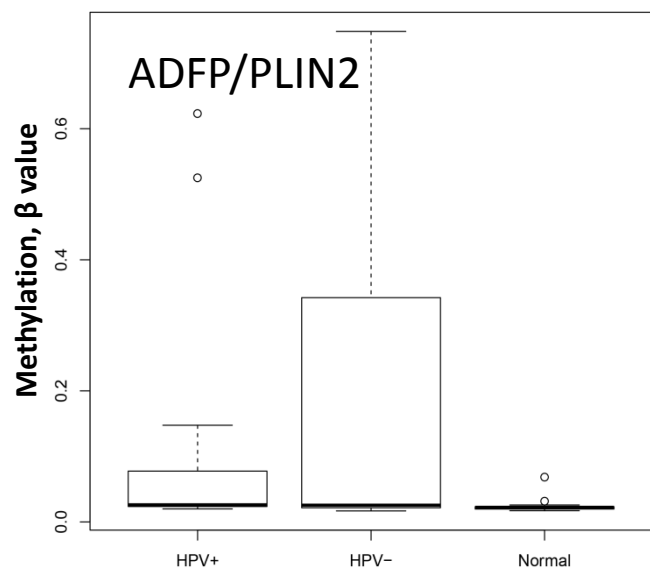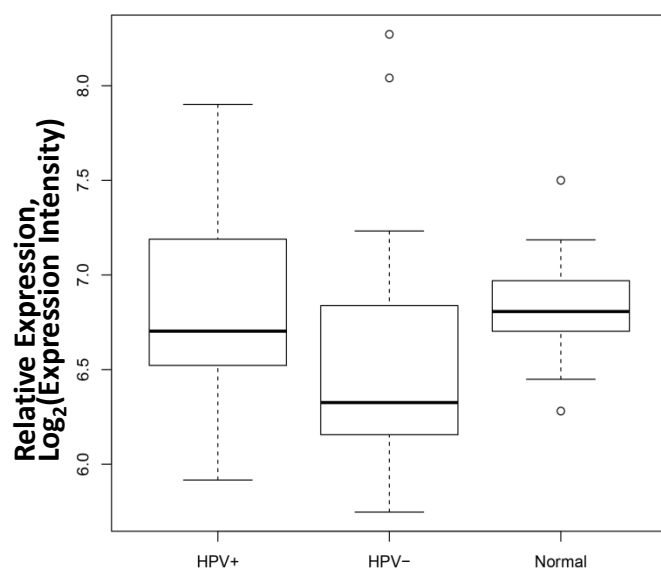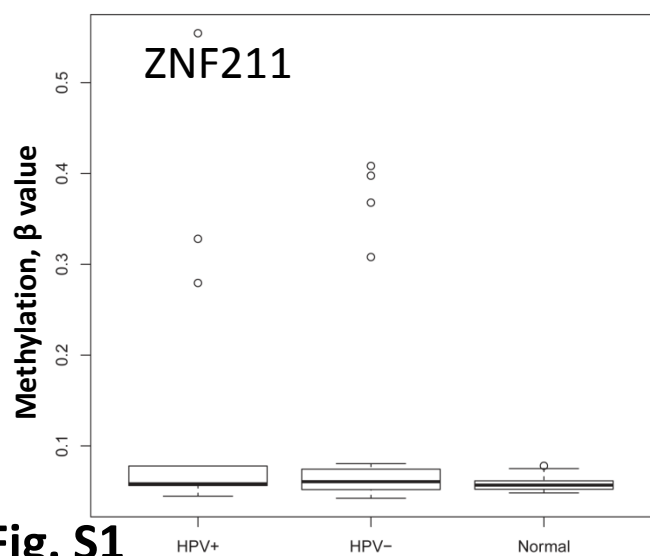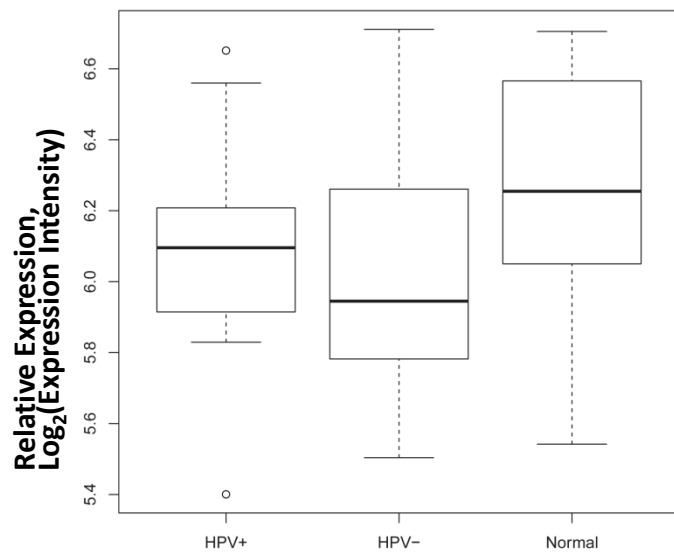

**Fig. S1**

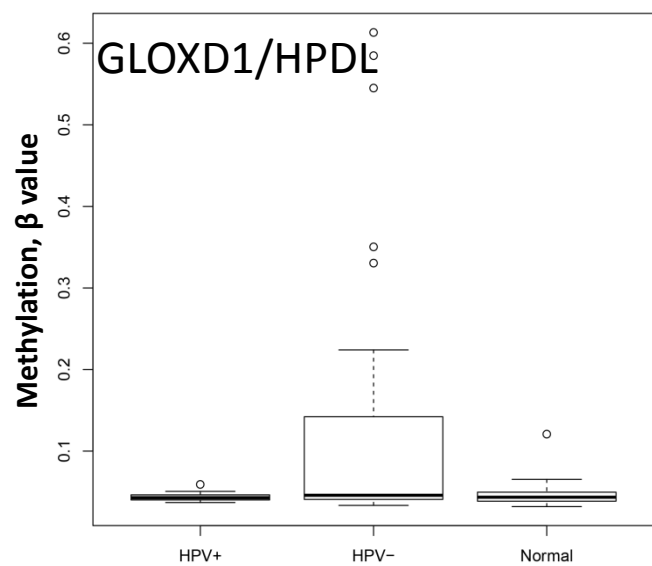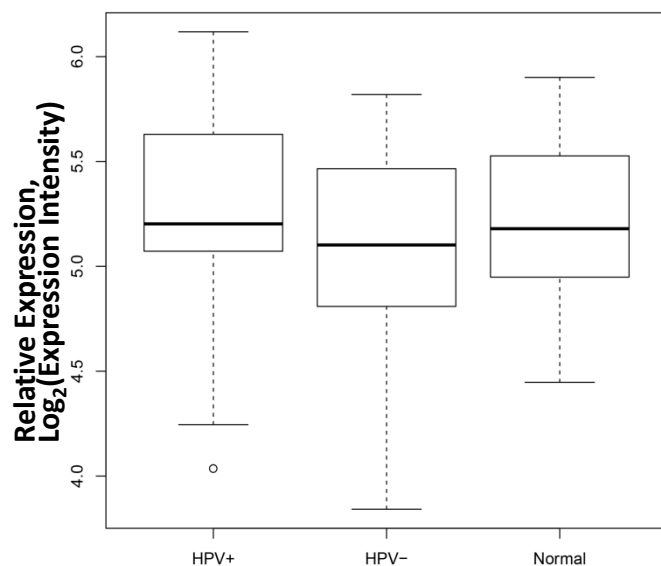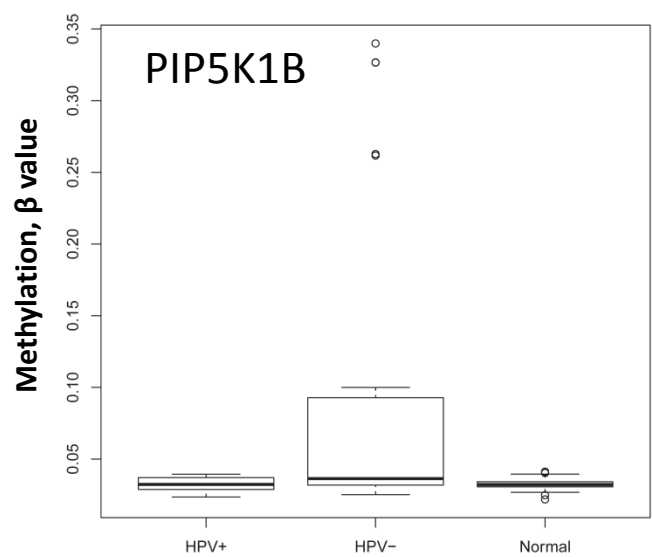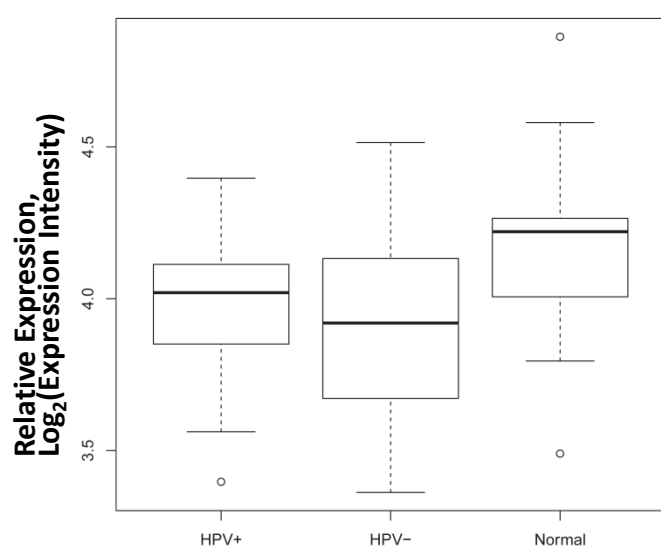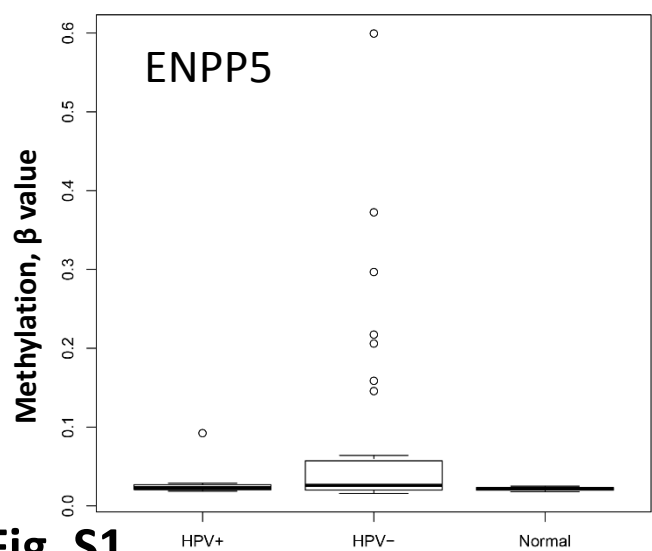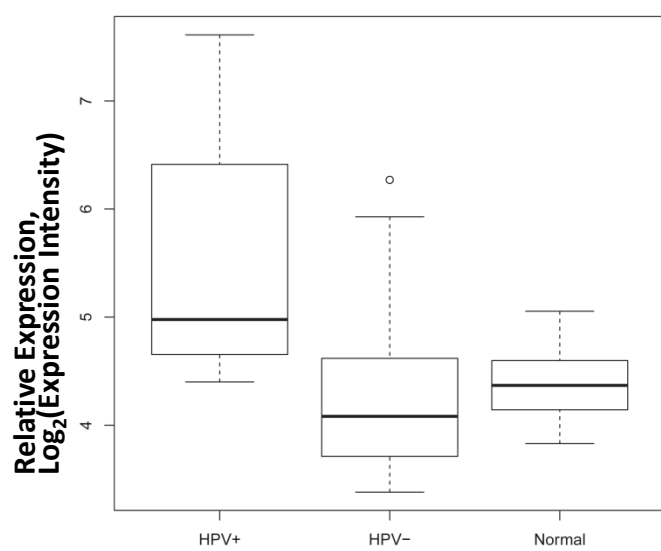

**Fig. S1**

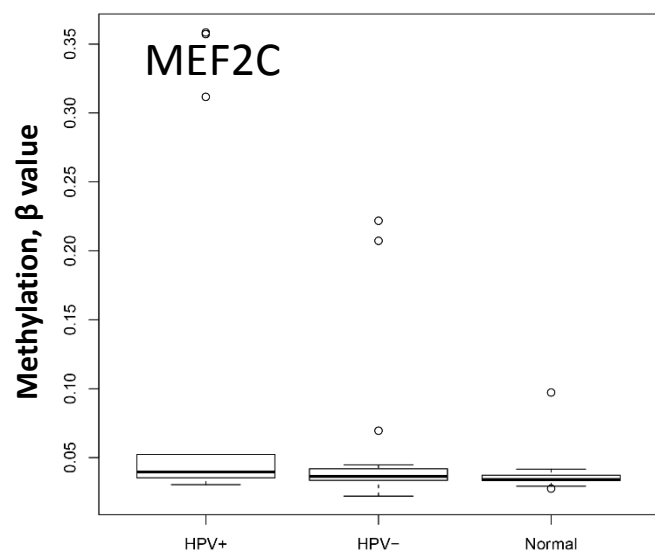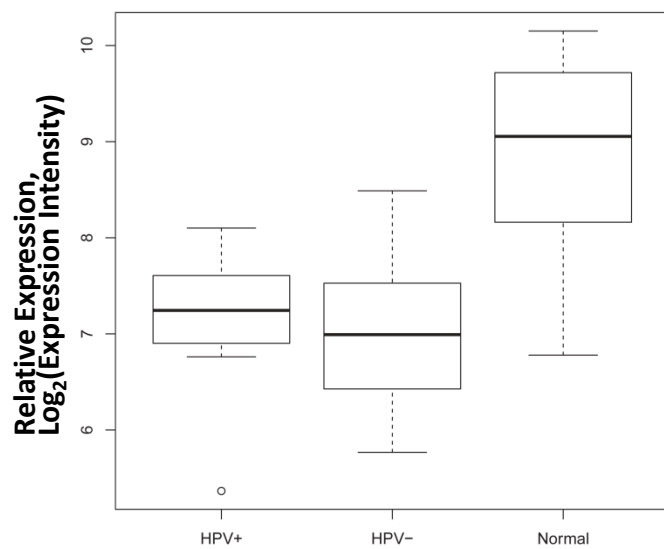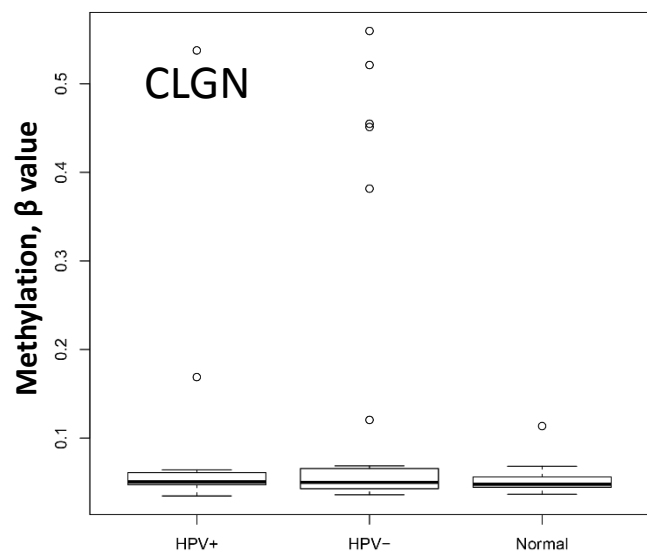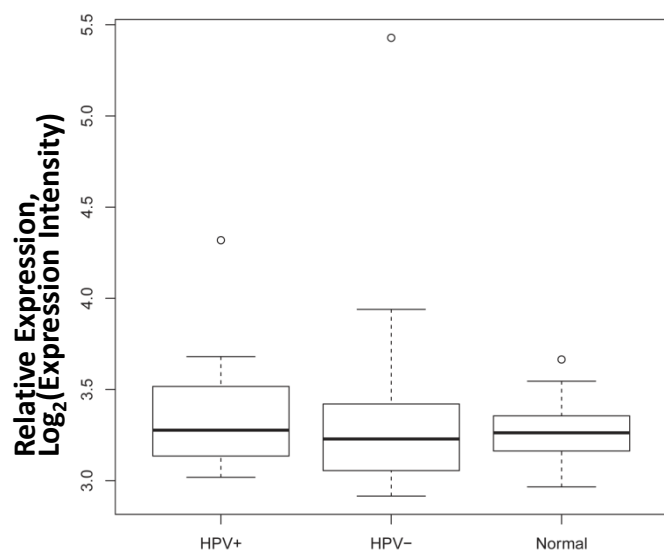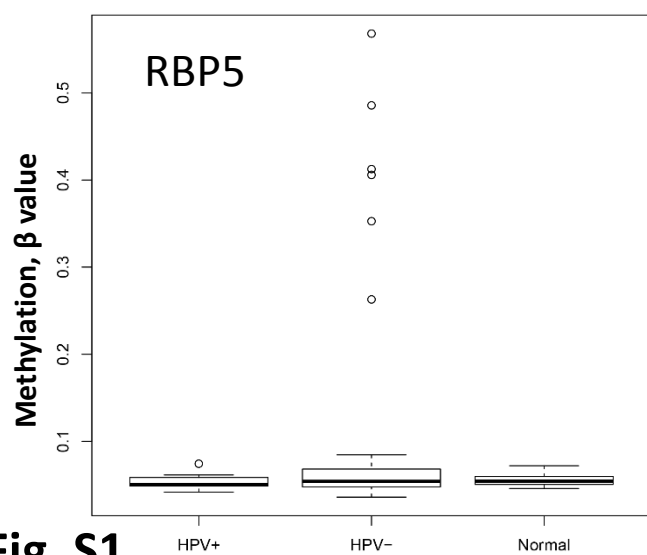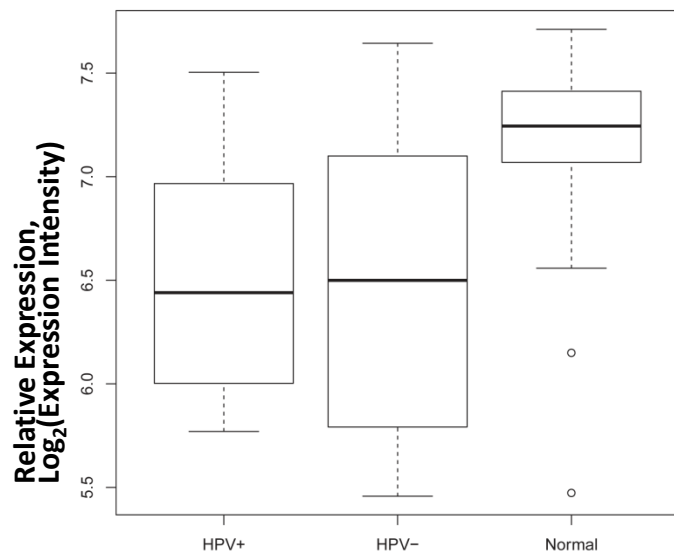

**Fig. S1**

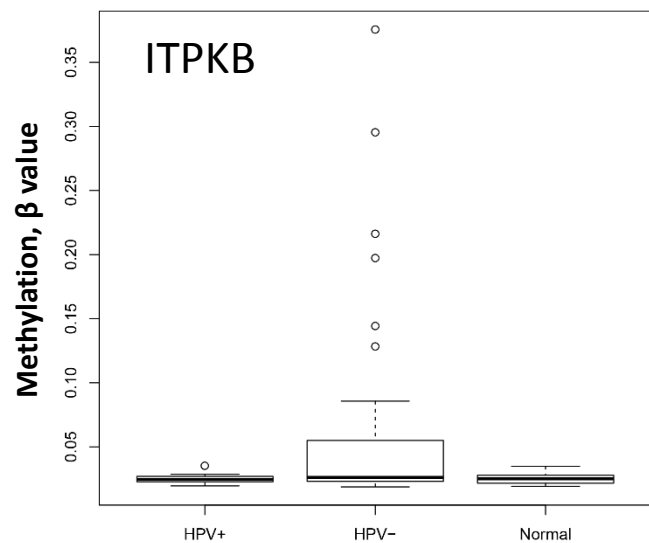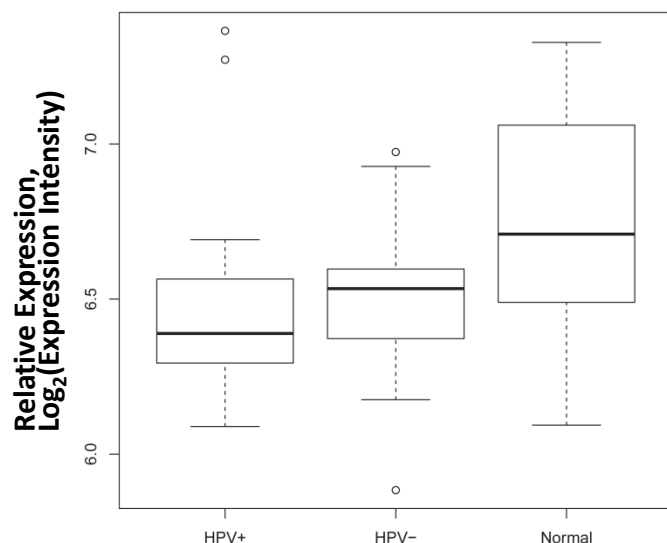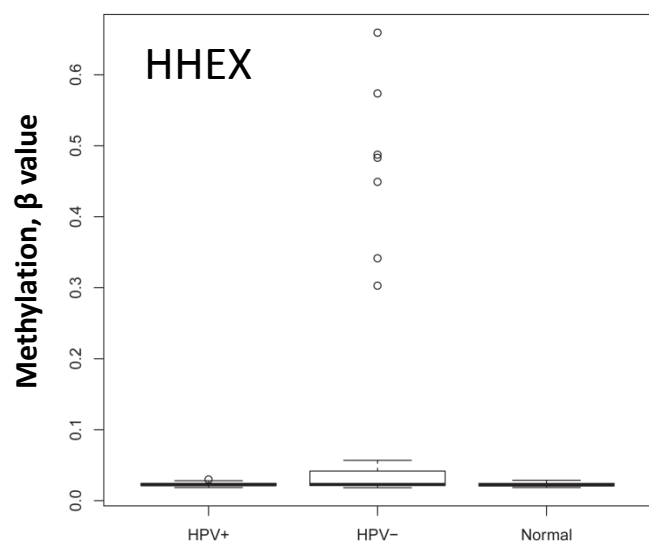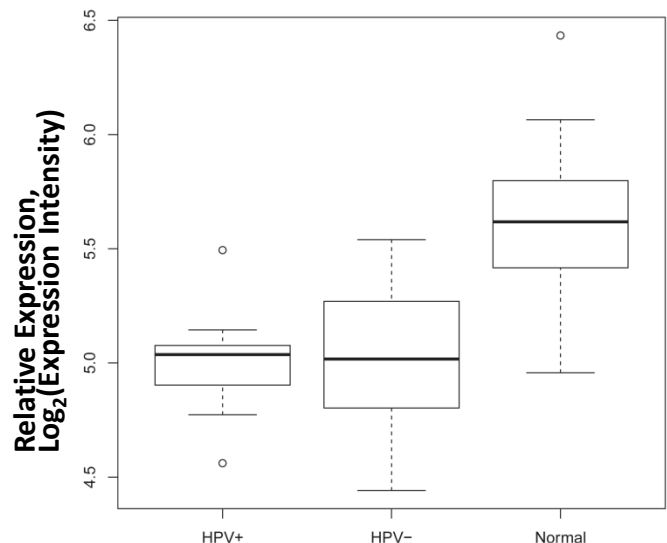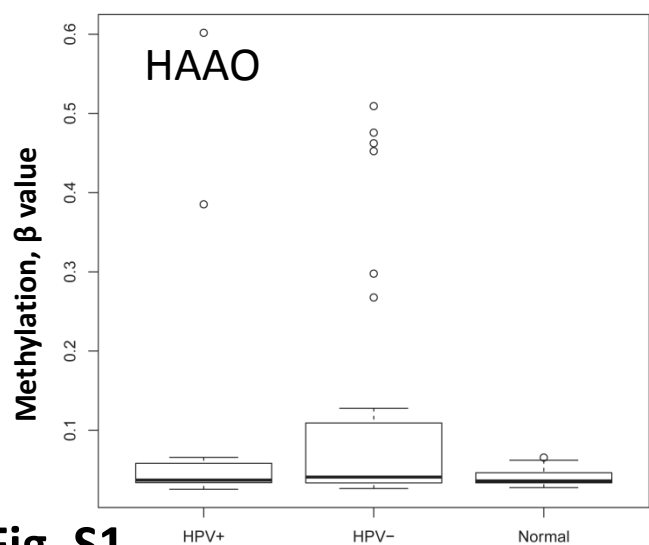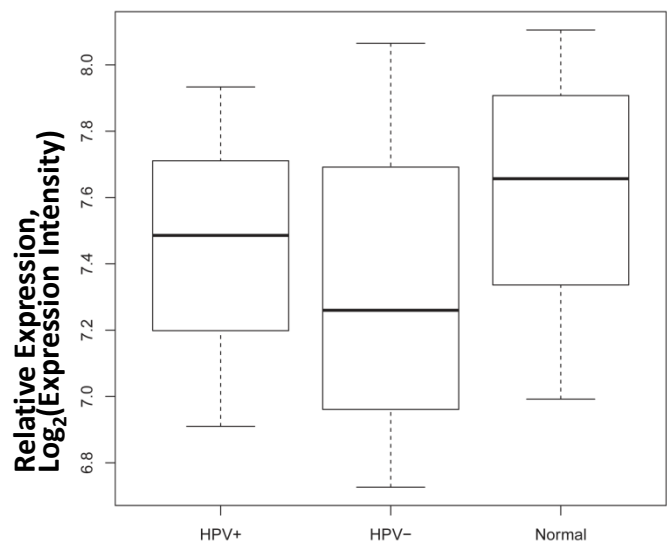

**Fig. S1**

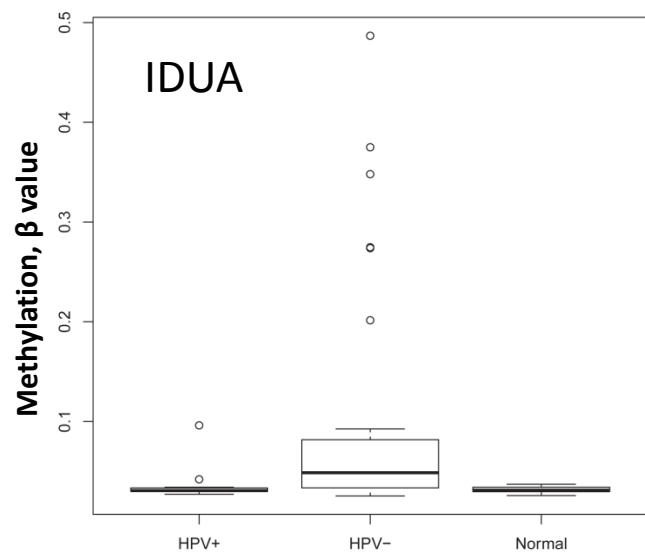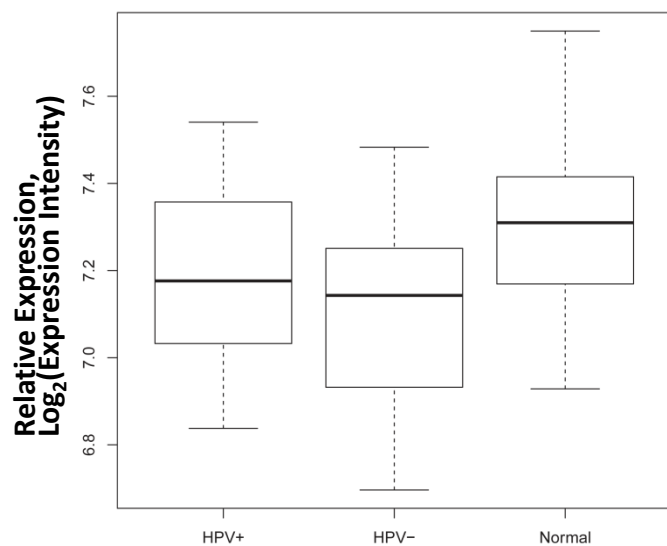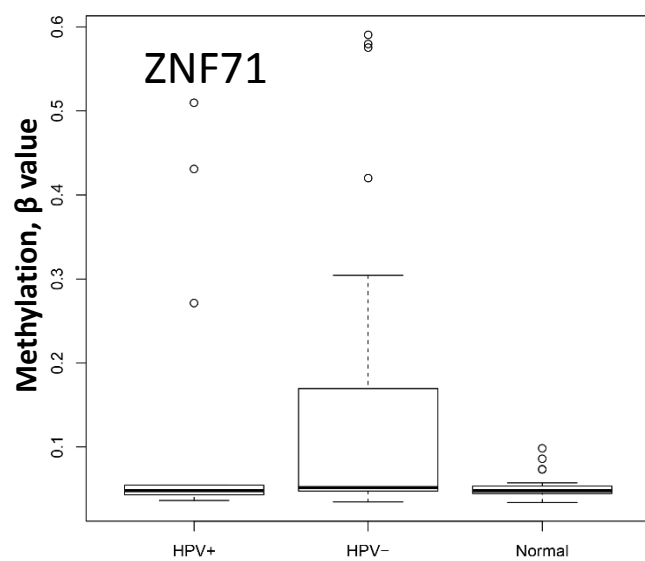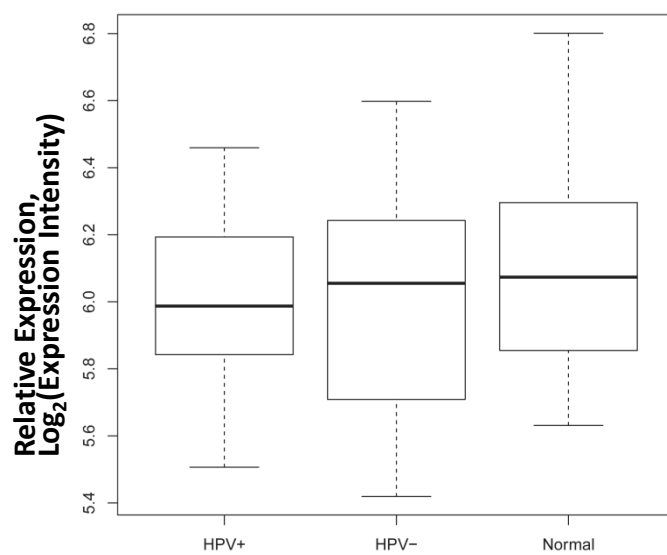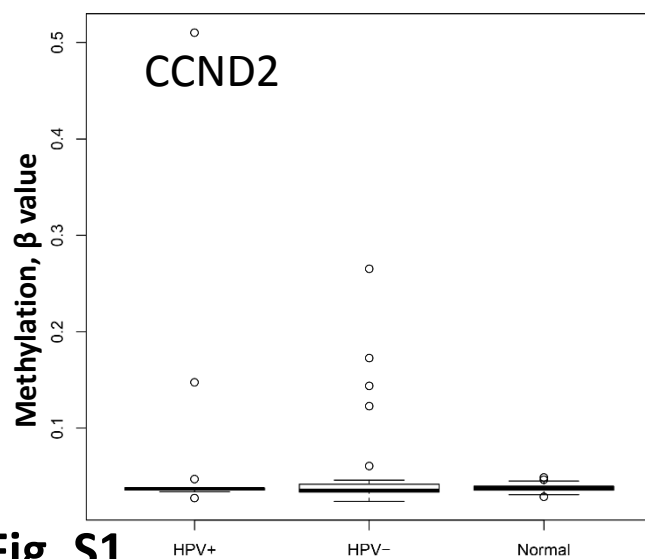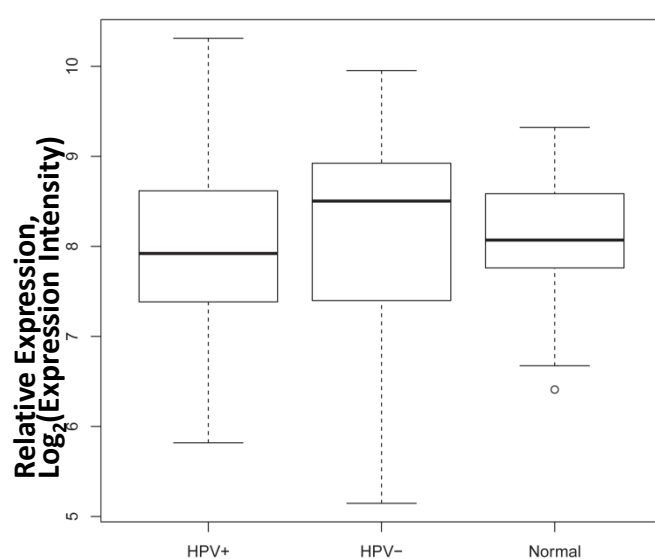

**Fig. S1**

Supplement: S1 Fig — B-values and expression intensity values were from Illumina Infinium HumanMethylation27 BeadChips and Affymetrix HuEx1.0 GeneChips arrays, respectively. HNSCC population was separated according to their HPV status to HPV- and HPV+ samples. The length of each box is the inter-quartile range and represents the middle 50% of the values. The horizontal line inside the box depicts the median. The lower and upper hinges of the box represent the 25th and 75th percentiles, respectively. The vertical dashed lines extend from the box to the upper and lower 1.5 inter-quartile values from the upper and lower hinges. The empty circles represent the outliers above and below the upper and lower hinges. The t-test p-values comparing group of patients for these 24 genes can be found in S5 and S6 Tables for methylation and expression values, respectively. (PDF) [file pone.0142148.s001.pdf]

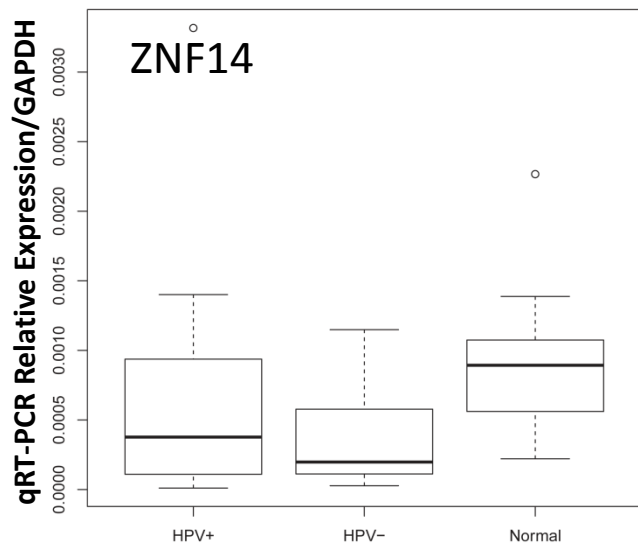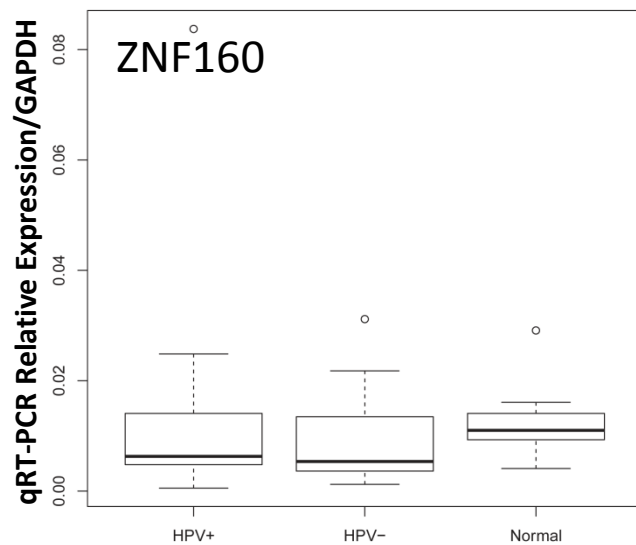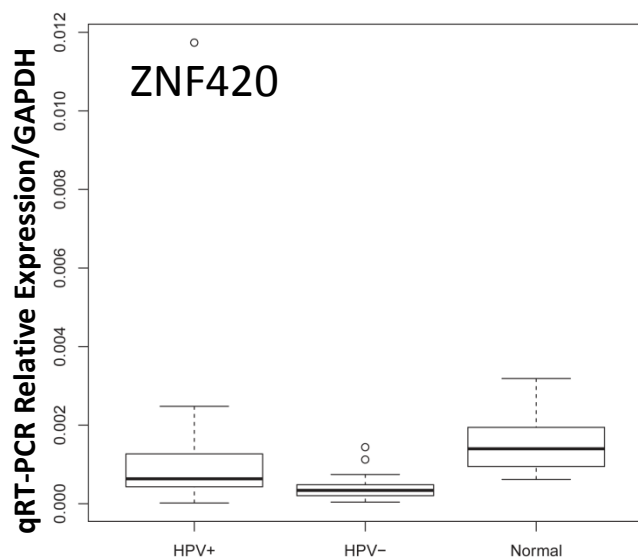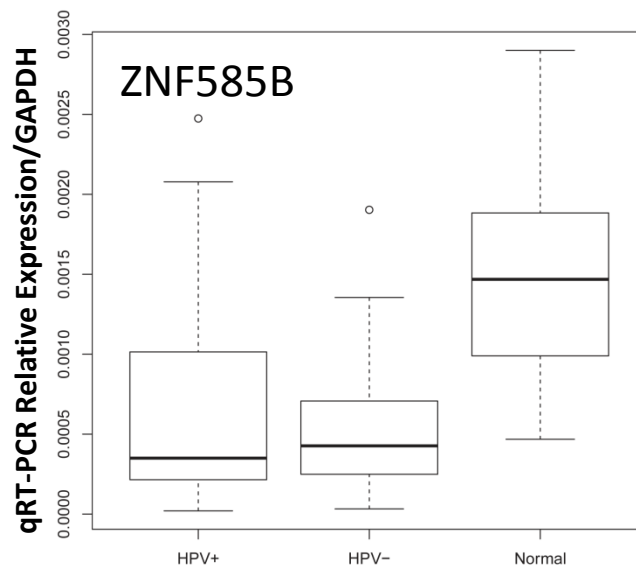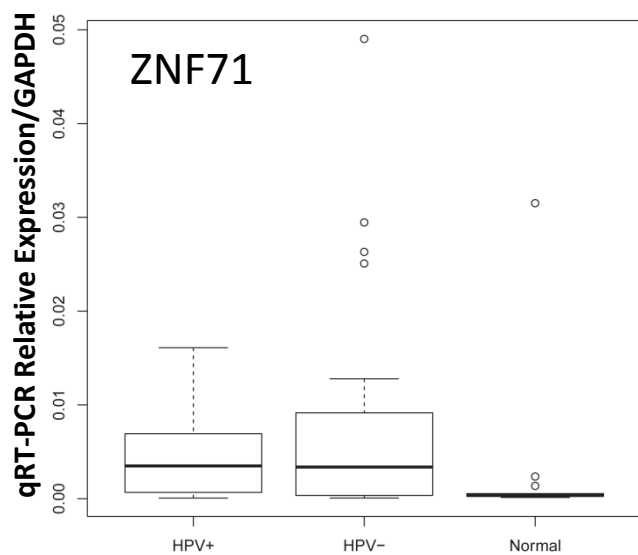

**Figure S2**

Supplement: S2 Fig — ZNF gene expression is shown relative to GAPDH. The boxplots were built as described for S1 Fig. Statistical analysis of the data can be found in S8 Table. (PDF) [file pone.0142148.s002.pdf]

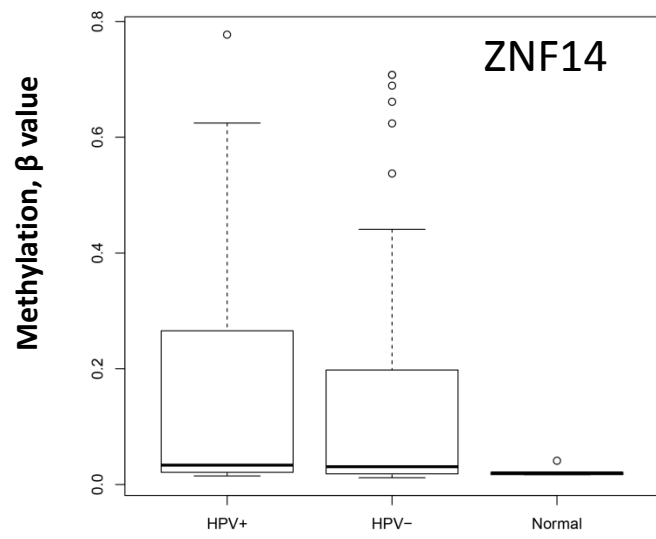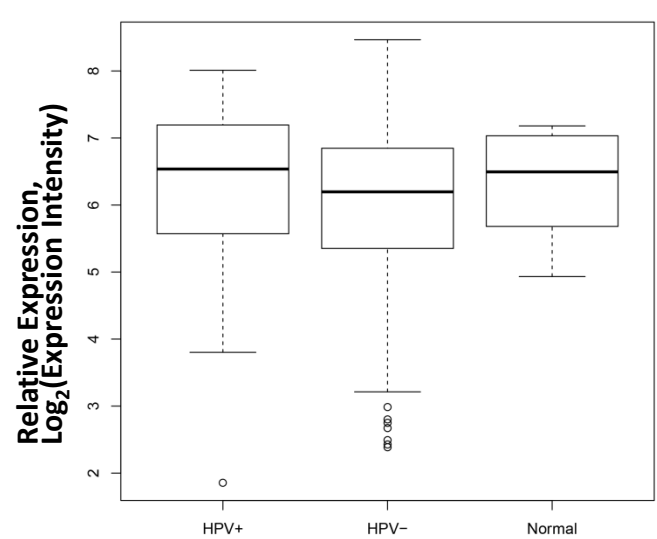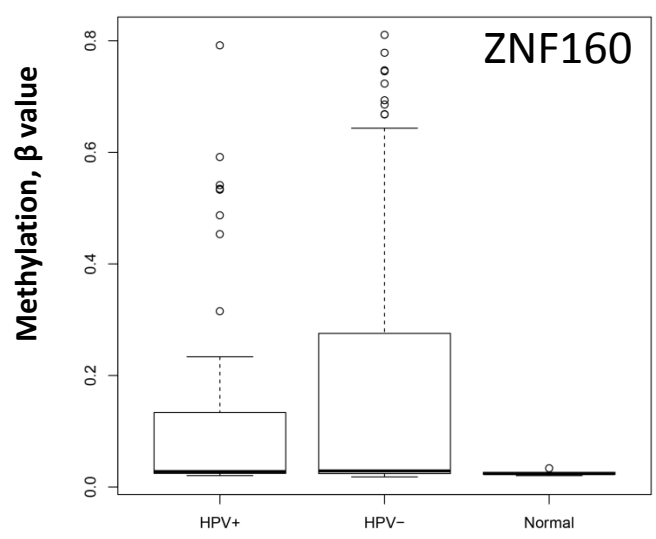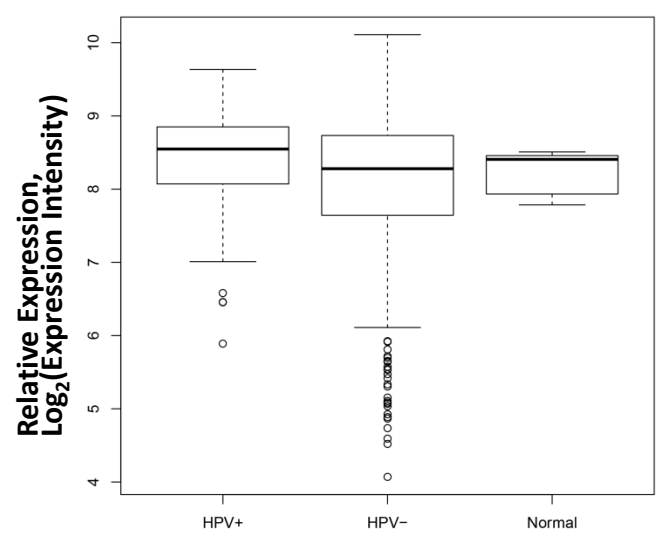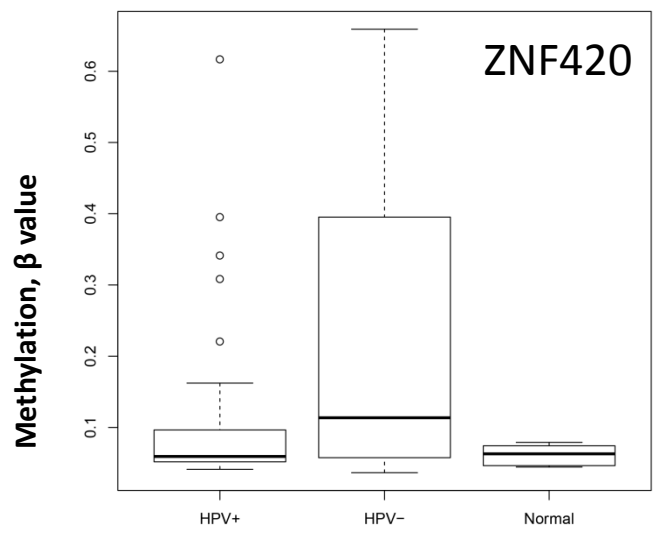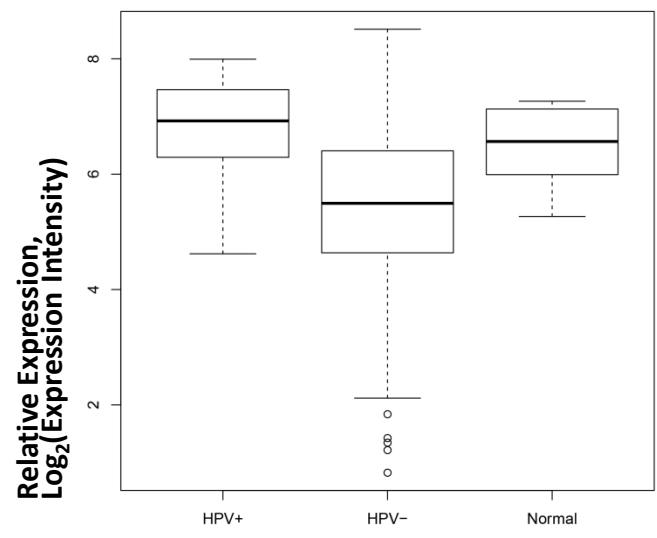

**Figure S3**

Supplement: S3 Fig — The boxplots were prepared as in S1 Fig. The t-test p-values comparing groups of patients for these 24 genes can be found in S10 and S11 Tables for methylation and expression values, respectively. (PDF) [file pone.0142148.s003.pdf]
