# Supplementary material for: Outlier Analysis Defines Zinc Finger Gene Family DNA Methylation in Tumors and Saliva of Head and Neck Cancer Patients
Source: PLoS One. 2015 Nov 6;10(11):e0142148. doi: 10.1371/journal.pone.0142148 (PMC4636259; doi:10.1371/journal.pone.0142148)
Supplement: S1 Table — (PDF) [file pone.0142148.s004.pdf]

Table S1. Clinical characteristics of HNSCC patients in the initial discovery cohort

	HNSCC (n = 44)	Normal samples (n = 25)
	n (%)	n (%)
Median age (range)	58±13 (45-80)	29±12 (18-65)
Male	32 (73%)	9 (36%)
Female	12 (27%)	16 (64%)
Race		
Caucasian	40 (91%)	14 (56%)
African American	3 (7%)	11 (44%)
Others	1 (2%)	
Smoking status		
Pack-years, mean (range)	39.7 (4-125)	29 (8-50)
Smokers	27 (61%)	3 (12%)
Non-smokers	12 (28%)	22 (88%)
Unknown	5 (11%)	
Drinking status		
Drink	25 (57%)	9 (36%)
Do not drink	12 (27%)	16 (64%)
Unknown	7 (16%)	
HPV16 positive	13 (30%)	
Tumor site		
Oral cavity	10 (23%)	
Oropharynx	17 (38%)	
Larynx	13 (30%)	
Hypopharynx	4 (9%)	
TNM stage		
I	5 (11%)	
II	2 (5%)	
III	5 (11%)	
IV	32 (73%)	
Disease status		
No evidence of disease	22 (50%)	
Alive with disease	1 (2%)	
Dead of disease	18 (41%)	
Dead of unrelated causes	3 (7%)	
