# Supplementary material for: Outlier Analysis Defines Zinc Finger Gene Family DNA Methylation in Tumors and Saliva of Head and Neck Cancer Patients
Source: PLoS One. 2015 Nov 6;10(11):e0142148. doi: 10.1371/journal.pone.0142148 (PMC4636259; doi:10.1371/journal.pone.0142148)
Supplement: S2 Table — (PDF) [file pone.0142148.s005.pdf]

Table S2. Clinical characteristics of HNSCC patients in the validation cohort

	HNSCC (n = 59)	Normal tissue (n = 31)	Normal saliva (n = 35)
	n (%)	n (%)	n (%)
**Median age (range)**	59±12 (35-87)	32±11 (18-57)	58±12 (32-77)
**Male**	47 (80%)	16 (52%)	12 (34%)
**Female**	12 (20%)	15 (48%)	23 (66%)
**Race**			
**Caucasian**	52 (88%)	16 (52%)	25 (71%)
**African American**	5 (9%)	12 (38%)	7 (20%)
**Others**	2 (3%)	3 (10%)	3 (9%)
**Smoking status**			
**Pack-years (range)**	43.1 (5-110)	73.9 (3-274)	280 (183-730)
**Smokers**	47 (80%)	9 (29%)	18 (51%)
**Non-smokers**	12 (20%)	22 (71%)	17 (49%)
**Drinking status**			
**Drink**	34 (58%)	4 (13%)	24 (69%)
**Do not drink**	19 (32%)	27 (87%)	11 (31%)
**Unknown**	6 (10%)		
**HPV16 positive**	18 (31%)		
**Tumor site**			
**Oral cavity**	15 (26%)		
**Oropharynx**	25 (42%)		
**Larynx**	17 (29%)		
**Hypopharynx**	2 (3%)		
**TNM stage**			
**I**	5 (9%)		
**II**	10 (17%)		
**III**	9 (15%)		
**IV**	32 (54%)		
**Unknown**	3 (5%)		
**Disease status**			
**No evidence of disease**	34 (58%)		
**Alive with disease**	7 (12%)		
**Dead of disease**	7 (12%)		
**Dead of unrelated causes**	11 (18%)		
