# Supplementary material for: Outlier Analysis Defines Zinc Finger Gene Family DNA Methylation in Tumors and Saliva of Head and Neck Cancer Patients
Source: PLoS One. 2015 Nov 6;10(11):e0142148. doi: 10.1371/journal.pone.0142148 (PMC4636259; doi:10.1371/journal.pone.0142148)
Supplement: S3 Table — (PDF) [file pone.0142148.s006.pdf]

Table S3. Primers, probes and expression assays used in the study

**Applied Biosystems-recommended expression assays**				
**No**	**Gene name**	**Assay ID**		
**1**	**ZNF14**	Hs00221420_m1		
**2**	**ZNF71**	Hs01934418_s1		
**3**	**ZNF160**	Hs00369142_m1		
**4**	**ZNF420**	Hs01557830_m1		
**5**	**ZNF585B**	Hs04189951_m1		
**6**	**GAPDH**	Hs02758991_g1		
**7**	**18S**	Hs99999901_s1		
**Quantitative Methylation-specific PCR (QMSP) primers and probes**				
**No**	**Gene name**	**Forward Primer**	**Reverse Primer**	**Probe**
**1**	**ZNF14**	GGATATCGTGATTTTTCGGACGTTG	CGACTACGAATCCAACTCCCACAA	6FAM/AAACCGAACTACGCCCGCGATAACC/TAMRA
**2**	**ZNF160**	GAAATCGTTTGAAATATTTACGTCGTT	AACGAAACTAAACGAAACACGTTA	6FAM/ACGATTTCGTATAATACCCACAACCCAACGC/TAMRA
**3**	**ZNF420**	GGTATGGTGTTCGGAGCGTT	CACGCGAAACCTCCAAATCT	6FAM/TAGAGGTATCGTTTTCGGAGCGTAGT/TAMRA
**4**	**β-actin**	TGGTGATGGAGGAGGTTTAGTAAGT	AACCAATAAAACCTACTCCTCCCTTAA	6FAM/ACCACCACCCAACACACAATAACAAACACA/TAMRA
**Bisulfite sequncing PCR primers**				
**No**	**Gene name**	**Forward Primer**	**Reverse Primer**	
1	**ADFP (PLIN2)**	GATTTTAGGTAGGGTTATTTTTAATTTTTA	CCAAACAAACCAAAAAACATTC	
2	**CCND2**	GGGTTGGTTATGGAGTTGTTG	AACATCCAAATAACCACCATTCTAC	
3	**CHFR**	GGATTTGTGTGATTTATTGTGTGTAAT	ACCATCTTTAATCCTAACCAAAC	
4	**CLGN**	GATTTGTAGGGGGAATTTTTTTT	AAAACCCAATCAAAACCCTAACT	
5	**ENPP5**	GGGGGTAATTTAGGTAGAAGTGATTAT	AATTATATTCCCAATTCCCAATCAT	
6	**FLJ22688 (FUZ)**	GGTTTTTTGGTTTTTTTTATTTTTT	TCCAAAACCCCACCTACTAAC	
7	**GLOXD1 (HPDL)**	GTAGTTATTGTGAGTTTTTGGGTTG	ACCTAAACTTATCCTTCTAAAACC	
8	**HAAO**	TTTTTAGATGGGAAAGTTAAATTTTGA	AAAAAATCCAAACCCTTCCTAAAC	
9	**HHEX**	GATTGGTTTTTTTGTATTTTGTAGG	ACACAAACCTATTAAACCAAATCCA	
10	**ICA1**	GGGTTGTAGGAAGTAGTAGGAGA	CTTATCAACAAATCAACCCTAAAC	
11	**IDUA**	GTTTTATTTAGGAGGTTGGGGTG	CAAAAACCTATACTCCTCCAAAAAC	
12	**ITPKB**	GGTTGTTTTGGATAGTTAATGTTTGTT	CCTACAAAACCCAAAAAAAACC	
13	**MEF2C**	AAGAGTGAAATTGATGATTTTTTTAGTT	ATACTTCTCCACCTAATTCAAACATACA	
14	**PIP5K1B**	GGGGTTTGTAGTTTTTTTAGT	CAACAAAAATACAAAACCCCTAAAC	
15	**RBP5**	TGGGGAGAAAGAAGTTAGAAGTTAG	CCTCCTTAAATCCCAAAACCT	
16	**RECK**	TTGAGGTTTTGGTTTGTTATTTAT	AAAACAAAAATTTCTCTCCTCAAAC	
17	**VILL**	TTTGGGGAAGTTTGTTTGAGA	ACTTACCCCATTCAAAAATATAAAC	
18	**ZNF14**	GTTATTGGATTTGTTTAATTAGGA	AAATTAACTACAAAAAAATCCCC	
19	**ZNF141**	GAGTTTGGGGAGGGAGATATATTT	TCCTCACAAAACCTAATTAAATACACA	
20	**ZNF160**	AGAGGAAAGTAGTTTGGTTTTTAAAATAAT	AACAAAAACCCCAAAAAAAA	
21	**ZNF211**	TGAAAATTTAAGATAGGGGTATTTT	CTCTCACTTAAAACTTAAAAATCTC	
22	**ZNF420**	GGGATAAGTAGGTTTTATAGGT	AAAATCCAAAATCTAACTCCC	
23	**ZNF585B**	TGGGTTGAAATTGGTTTTTAAGT	TAACTAACCCTACAAACCCTCAATC	
24	**ZNF71**	GTTTTTTGTGAGATGGAGGAGTTTA	CTACCTATCTCTCACACAAACCAC	
