# Supplementary material for: Outlier Analysis Defines Zinc Finger Gene Family DNA Methylation in Tumors and Saliva of Head and Neck Cancer Patients
Source: PLoS One. 2015 Nov 6;10(11):e0142148. doi: 10.1371/journal.pone.0142148 (PMC4636259; doi:10.1371/journal.pone.0142148)
Supplement: S4 Table — (PDF) [file pone.0142148.s007.pdf]

**Table S4. Outlier scores for 37 candidates from the methylation array**

| No | Gene name | Outlier score |
|---|---|---|
| 1 | **HHEX** | 67.51054185 |
| 2 | **VILL** | 47.65082835 |
| 3 | **CHFR** | 37.23973321 |
| 4 | **ZNF160** | 36.2084266 |
| 5 | **MPDU1** | 31.13417209 |
| 6 | **ZNF134** | 28.9373152 |
| 7 | **BSG** | 28.1162042 |
| 8 | **FLJ22688 (FUZ)** | 28.08415274 |
| 9 | **CLSTN3** | 27.69596653 |
| 10 | **RBP5** | 24.39698918 |
| 11 | **MEF2C** | 23.479971 |
| 12 | **CLGN** | 23.40885042 |
| 13 | **IDUA** | 21.7933596 |
| 14 | **PIP5K1B** | 20.67732627 |
| 15 | **ADFP (PLIN2)** | 19.83258688 |
| 16 | **ZNF420** | 19.46419102 |
| 17 | **ZNF141** | 19.02108471 |
| 18 | **RASA4** | 18.80958368 |
| 19 | **KCNQ1** | 18.67906133 |
| 20 | **RAB39** | 17.85805985 |
| 21 | **ZNF211** | 17.77623482 |
| 22 | **RHOF** | 17.05440732 |
| 23 | **ENPP5** | 16.47452634 |
| 24 | **ZNF71** | 16.36372914 |
| 25 | **CCND2** | 16.2578808 |
| 26 | **GLOXD1 (HPDL)** | 16.18834852 |
| 27 | **ICA1** | 15.63623555 |
| 28 | **ZNF14** | 15.4458021 |
| 29 | **HAAO** | 15.41014583 |
| 30 | **SLC8A3** | 15.18526618 |
| 31 | **RECK** | 15.04356674 |
| 32 | **ITPKB** | 14.60792797 |
| 33 | **PFKFB4** | 14.52909531 |
| 34 | **ZNF585B** | 14.41258458 |
| 35 | **JAM2** | 13.49463275 |
| 36 | **HIST1H3I** | 13.47289421 |
| 37 | **RHOF** | 13.21077435 |
