# Supplementary material for: Outlier Analysis Defines Zinc Finger Gene Family DNA Methylation in Tumors and Saliva of Head and Neck Cancer Patients
Source: PLoS One. 2015 Nov 6;10(11):e0142148. doi: 10.1371/journal.pone.0142148 (PMC4636259; doi:10.1371/journal.pone.0142148)
Supplement: S5 Table — These groups were compared by t-test. (PDF) [file pone.0142148.s008.pdf]

Table S5. DNA methylation β-values in different patient groups in the discovery cohort (Illumina Methylation array 27). These groups were compared by t-test

		mean values				t-test p-values			
gene name	probe name	Normal	HPV+ HNSCC	HPV- HNSCC	HNSCC samples	Normal vs HNSCC	Normal vs HPV- HNSCC	Normal vs HPV+ HNSCC	HPV+ vs HPV- HNSCC
ADFP/PLIN2	cg13060646	0.023728	0.125001	0.181365	0.164712	**0.000262**	**0.001336**	0.098238	0.439916
CCND2	cg02765328	0.038041	0.081599	0.053888	0.062075	0.063523	0.10312	0.259029	0.477457
CHFR	cg23653008	0.036703	0.035878	0.132738	0.10412	**0.003886**	**0.003216**	0.556439	**0.002995**
CLGN	cg21246783	0.05153	0.095938	0.119633	0.112632	**0.011681**	**0.025483**	0.266268	0.623467
ENPP5	cg04006554	0.021809	0.028622	0.084132	0.067731	**0.010324**	**0.013286**	0.231449	**0.028826**
FUZ/FLJ22688	cg10971790	0.028476	0.028926	0.142187	0.108723	**0.002146**	**0.001794**	0.666406	**0.001861**
GLOXD1/HPDL	cg26781150	0.046978	0.044124	0.132597	0.106458	**0.011834**	**0.009428**	0.464485	**0.007331**
HAAO	cg01561916	0.040795	0.109611	0.121982	0.118327	**0.00224**	**0.005891**	0.185261	0.827525
HHEX	cg11378840	0.022472	0.023113	0.124658	0.094656	**0.007979**	**0.007441**	0.557335	**0.007786**
ICA1	cg05434957	0.038339	0.040001	0.110369	0.089578	**0.016862**	**0.016628**	0.733295	**0.019617**
IDUA	cg04912050	0.031384	0.03681	0.102325	0.082969	**0.002303**	**0.00265**	0.306356	**0.005816**
ITPKB	cg01259619	0.025203	0.025307	0.068049	0.055421	**0.012544**	**0.011712**	0.939866	**0.01195**
MEF2C	cg08223748	0.037108	0.108545	0.048646	0.066344	**0.02975**	0.182593	0.078907	0.138837
PIP5K1B	cg21413251	0.03271	0.03265	0.087472	0.071274	**0.005419**	**0.004724**	0.971547	**0.004721**
RBP5	cg15569340	0.055648	0.053663	0.123677	0.102991	**0.020827**	**0.018162**	0.469516	**0.015486**
RECK	cg12717594	0.031696	0.164234	0.164364	0.164326	**0.000182**	**0.002164**	**0.041781**	0.998542
VILL	cg26113512	0.034087	0.022532	0.101018	0.077829	0.063519	**0.033231**	0.278204	**0.009787**
ZNF14	cg13714039	0.02519	0.14928	0.199162	0.184424	**1.61E-05**	**0.000137**	0.055819	0.48829
ZNF141	cg13108181	0.046357	0.046596	0.115592	0.095207	**0.032337**	**0.031699**	0.89725	**0.032334**
ZNF160	cg03712038	0.026017	0.077667	0.113692	0.103048	**0.003597**	**0.007178**	0.270825	0.511796
ZNF211	cg05908775	0.058415	0.133493	0.09941	0.10948	**0.00887**	**0.042609**	0.110102	0.483606
ZNF420	cg03391040	0.038961	0.083816	0.19823	0.164426	**0.000249**	**0.000447**	0.25885	**0.045561**
ZNF585B	cg03751813	0.043149	0.134508	0.120277	0.124481	**0.000995**	**0.004183**	0.105041	0.808074
ZNF71	cg15705469	0.05286	0.128403	0.142718	0.138489	**0.001748**	**0.007409**	0.124985	0.798173

significant p-values are bolded
