# Supplementary material for: Outlier Analysis Defines Zinc Finger Gene Family DNA Methylation in Tumors and Saliva of Head and Neck Cancer Patients
Source: PLoS One. 2015 Nov 6;10(11):e0142148. doi: 10.1371/journal.pone.0142148 (PMC4636259; doi:10.1371/journal.pone.0142148)
Supplement: S6 Table — These groups were compared by t-test. (PDF) [file pone.0142148.s009.pdf]

Table S6. Gene expression values in different patient groups in the discovery cohort (Affymetrix Exon array). These groups were compared by t-test

gene name	mean values				t-test p-values			
Gene name	Normal	HPV+ HNSCC	HPV- HNSCC	HNSCC samples	Normal vs HNSCC	Normal vs HPV- HNSCC	Normal vs HPV+ HNSCC	HPV+ vs HPV- HNSCC
ADFP/PLIN2	6.828791	6.856795	6.53223	6.628124	0.052706	**0.015147**	0.857517	0.081213
CCND2	7.964527	8.050868	8.178248	8.140613	0.457123	0.415837	0.820005	0.752144
CHFR	6.910373	6.740622	6.604958	6.645041	**3.56E-06**	**1.15E-05**	**0.017986**	0.095736
CLGN	3.249259	3.356431	3.290879	3.310247	0.402197	0.639663	0.32413	0.614905
ENPP5	4.300537	5.39756	4.216863	4.565705	0.101151	0.55456	**0.002363**	**0.00149**
FUZ/FLJ22688	6.61526	6.445678	6.402016	6.414916	**0.001468**	**0.002182**	0.074981	0.646842
GLOXD1/HPDL	5.200047	5.181173	5.034794	5.078043	0.277031	0.164427	0.923122	0.46951
HAAO	7.583502	7.428122	7.318344	7.350778	**0.019405**	**0.017599**	0.214384	0.3903
HHEX	5.570432	4.980735	5.016552	5.005969	**1.88E-08**	**9.64E-08**	**4.66E-07**	0.673576
ICA1	5.093469	5.229217	4.699842	4.856248	**0.01566**	**0.000193**	0.39712	**0.005228**
IDUA	7.293496	7.20566	7.095098	7.127765	**0.002894**	**0.000933**	0.259645	0.154558
ITPKB	6.72757	6.512236	6.502931	6.50568	**0.010936**	**0.010236**	0.097697	0.933748
MEF2C	8.771538	7.19093	7.046082	7.088878	**8.91E-09**	**1.24E-08**	**2.4E-06**	0.553483
PIP5K1B	4.129184	3.927102	3.875381	3.890662	**0.001217**	**0.001761**	**0.042869**	0.593242
RBP5	7.152075	6.494937	6.461107	6.471102	**6.68E-06**	**5.49E-05**	**0.001553**	0.864491
RECK	5.981852	5.549445	5.564853	5.560301	**7.86E-07**	**2.39E-05**	**0.002396**	0.910083
VILL	6.45837	6.322891	6.329111	6.327273	**0.04139**	0.077064	0.158377	0.951213
ZNF14	4.247603	4.157066	3.988908	4.038591	**0.008668**	**0.003019**	0.418598	0.146461
ZNF141	7.231162	6.983735	6.464232	6.617722	**0.002752**	**0.000393**	0.326989	**0.028322**
ZNF160	6.221478	6.213092	6.09809	6.132068	0.289912	0.202295	0.950689	0.447613
ZNF211	6.330632	6.150811	6.089739	6.107783	**0.006786**	**0.007072**	0.116435	0.582587
ZNF420	5.429231	5.474566	5.134933	5.235279	**0.007806**	**0.000304**	0.668402	**0.00692**
ZNF585B	5.7198	5.754026	5.172979	5.344652	**0.006026**	**0.00013**	0.873699	**0.014521**
ZNF71	6.067581	6.002662	6.02152	6.015948	0.495626	0.580522	0.521764	0.851647

significant p-values are bolded
