# Supplementary material for: Outlier Analysis Defines Zinc Finger Gene Family DNA Methylation in Tumors and Saliva of Head and Neck Cancer Patients
Source: PLoS One. 2015 Nov 6;10(11):e0142148. doi: 10.1371/journal.pone.0142148 (PMC4636259; doi:10.1371/journal.pone.0142148)
Supplement: S7 Table — (PDF) [file pone.0142148.s010.pdf]

Table S7. Bisulfite sequencing of the promoter DNA hypermethylation detection in primary tissues from HNSCC and non-cancerous patients of the validation cohort

	Percentage of patients with hemi or fully methylated promoters, detected in primary tumor tissues	Percentage of patients with hemi or fully methylated promoters, detected in normal tissues	Fisher's exact test
	**%**	**%**	**p-value**
**ZNF14**	43.8	0	**<0.0001**
**ZNF141**	9.4	78.6	**<0.0001**
**ZNF160**	46.9	14.3	**0.0011**
**ZNF211**	0.0	0	ND
**ZNF420**	25.0	0	**0.0018**
**ZNF585B**	34.4	0	**<0.0001**
**ZNF71**	37.5	0	**<0.0001**
