# Supplementary material for: Outlier Analysis Defines Zinc Finger Gene Family DNA Methylation in Tumors and Saliva of Head and Neck Cancer Patients
Source: PLoS One. 2015 Nov 6;10(11):e0142148. doi: 10.1371/journal.pone.0142148 (PMC4636259; doi:10.1371/journal.pone.0142148)
Supplement: S8 Table — These groups were compared by t-test. (PDF) [file pone.0142148.s011.pdf]

Table S8. QRT-PCR gene expression values in different patient groups in the validation cohort. These groups were compared by t-test

	mean values				t-test p-values			
Gene name	Normal	HPV+ HNSCC	HPV- HNSCC	HNSCC samples	Normal vs HNSCC	Normal vs HPV- HNSCC	Normal vs HPV+ HNSCC	HPV+ vs HPV- HNSCC
ZNF14	0.000895	0.007676	0.000363	0.002877	0.259254	**3.21E-05**	0.184195	0.155933
ZNF71	0.002763	0.004603	0.008562	0.007201	**0.03605**	**0.004767**	0.100092	0.096148
ZNF160	0.012099	0.012958	0.009221	0.010506	0.399782	0.181758	0.675446	0.311006
ZNF420	0.001563	0.000724	0.000421	0.000525	**5.58E-08**	**6.72E-09**	**0.000432**	0.077166
ZNF585B	0.00143	0.000567	0.000551	0.000557	**7.55E-08**	**7.73E-08**	**2.41E-05**	0.915036

Significant p-values are bolded
