# Supplementary material for: Outlier Analysis Defines Zinc Finger Gene Family DNA Methylation in Tumors and Saliva of Head and Neck Cancer Patients
Source: PLoS One. 2015 Nov 6;10(11):e0142148. doi: 10.1371/journal.pone.0142148 (PMC4636259; doi:10.1371/journal.pone.0142148)
Supplement: S9 Table — (PDF) [file pone.0142148.s012.pdf]

Table S9. Comparison of the promoter DNA hypermethylation detection in all primary tissues from HNSCC and non-cancerous patients from three cohorts using four different DNA methylation detection techniques

		Discovery Cohort	Validation Cohort		TCGA
		**DNA methylation array 27K**	**Bisulfite Sequensing**	**QMSP**	**DNA methylation array 450**
		**%**	**%**	**%**	**%**
**ZNF14**	Detection in tumor samples	41	43.8	44.1	40.5
	Detection in normal	0	0	0	0
**ZNF160**	Detection in tumor samples	25	46.9	39	34.8
	Detection in normal	0	14.3	0	0
**ZNF420**	Detection in tumor samples	34	25	32.2	48
	Detection in normal	0	0	0	0

For the array data the detection cut-off for each gene was set as normal mean plus 3 standard deviations. For QMSP data the cut of was DNA methylation detection at 38 PCR cycles.
