# Supplementary material for: Outlier Analysis Defines Zinc Finger Gene Family DNA Methylation in Tumors and Saliva of Head and Neck Cancer Patients
Source: PLoS One. 2015 Nov 6;10(11):e0142148. doi: 10.1371/journal.pone.0142148 (PMC4636259; doi:10.1371/journal.pone.0142148)
Supplement: S10 Table — These groups were compared by t-test. (PDF) [file pone.0142148.s013.pdf]

Table S10. DNA methylation β-values in different patient groups in the TCGA-HNSCC cohort (RNA-Seq). These groups were compared by t-test

		mean values				t-test p-values			
gene name	probe name	Normal	HPV+ HNSCC	HPV- HNSCC	HNSCC samples	Normal vs HNSCC	Normal vs HPV- HNSCC	Normal vs HPV+ HNSCC	HPV+ vs HPV- HNSCC
ZNF14	cg17476421	0.022708	0.165640595	0.116051	0.12245	**2.61E-16**	**2.61E-16**	**0.000267**	0.18017
ZNF160	cg08228914	0.025282	0.144364065	0.151199	0.150317	**3.88E-18**	**3.88E-18**	**0.002679**	0.862113
ZNF420	cg14340610	0.061692	0.110454937	0.221476	0.207151	**3.5E-21**	**3.5E-21**	**0.027134**	**1.64E-05**

significant p-values are bolded
