# Supplementary material for: Outlier Analysis Defines Zinc Finger Gene Family DNA Methylation in Tumors and Saliva of Head and Neck Cancer Patients
Source: PLoS One. 2015 Nov 6;10(11):e0142148. doi: 10.1371/journal.pone.0142148 (PMC4636259; doi:10.1371/journal.pone.0142148)
Supplement: S11 Table — These groups were compared by t-test. (PDF) [file pone.0142148.s014.pdf]

Table S11. Gene expression values in different patient groups in the TCGA-HNSCC cohort (RNA-Seq). These groups were compared by t-test

	mean values				t-test p-values			
Gene name	Normal	HPV+ HNSCC	HPV- HNSCC	HNSCC samples	Normal vs HNSCC	Normal vs HPV- HNSCC	Normal vs HPV+ HNSCC	HPV+ vs HPV- HNSCC
ZNF14	6.302398	6.282542	6.000884	6.037227	0.462655	0.462655	0.964653	0.241017
ZNF160	8.249721	8.286613	7.965842	8.007232	0.085052	0.085052	0.849408	0.055017
ZNF420	6.464059	6.772611	5.361683	5.543738	**0.014613**	**0.014613**	0.393456	**4.21E-12**

Significant p-values are bolded
